# Tiny Bird, Huge Mystery—The Possibly Extinct Hooded Seedeater (*Sporophila melanops*) Is a Capuchino with a Melanistic Cap

**DOI:** 10.1371/journal.pone.0154231

**Published:** 2016-05-11

**Authors:** Juan Ignacio Areta, Vítor de Q. Piacentini, Elisabeth Haring, Anita Gamauf, Luís Fábio Silveira, Erika Machado, Guy M. Kirwan

**Affiliations:** 1 Instituto de Bio y Geociencias del Noroeste Argentino (IBIGEO-CONICET), Rosario de Lerma, Salta, Argentina; 2 Museu de Zoologia da Universidade de São Paulo (MZUSP), Ipiranga, São Paulo, Brasil; 3 Ornithology Department, Academy of Natural Sciences of Drexel University, Philadelphia, United States of America; 4 Museum of Natural History Vienna, Vienna, Austria; 5 Department of Integrative Zoology, University of Vienna, Vienna, Austria; 6 Field Museum of Natural History, Chicago, United States of America; University of Veterinary Medicine Hanover, GERMANY

## Abstract

Known with certainty solely from a unique male specimen collected in central Brazil in the first quarter of the 19th century, the Critically Endangered (Possibly Extinct) Hooded Seedeater *Sporophila melanops* has been one of the great enigmas of Neotropical ornithology, arguably the only one of a host of long-lost species from Brazil to remain obstinately undiscovered. We reanalysed the morphology of the type specimen, as well as a female specimen postulated to represent the same taxon, and sequenced mitochondrial DNA (COI and Cyt-b) from both individuals. Furthermore, we visited the type locality, at the border between Goiás and Mato Grosso, and its environs on multiple occasions at different seasons, searching for birds with similar morphology to the type, without success. Novel genetic and morphological evidence clearly demonstrates that the type of *S*. *melanops* is not closely related to Yellow-bellied Seedeater *S*. *nigricollis*, as has been frequently postulated in the literature, but is in fact a representative of one of the so-called capuchinos, a clade of attractively plumaged seedeaters that breed mostly in the Southern Cone of South America. Our morphological analysis indicates that *S*. *melanops* has a hitherto unreported dark-coffee throat and that it is probably a Dark-throated Seedeater *S*. *ruficollis* collected within its wintering range, acquiring breeding plumage and showing melanism on the cap feathers. Alternatively, it may be a melanistic-capped individual of a local population of seedeaters known to breed in the Esteros del Iberá, Corrientes, Argentina, to which the name *S*. *ruficollis* might be applicable, whilst the name *S*. *plumbeiceps* might be available for what is currently known as *S*. *ruficollis*. A hybrid origin for *S*. *melanops* cannot be ruled out from the available data, but seems unlikely. The purported female specimen of *S*. *melanops* pertains either to *S*. *nigricollis* or to Double-collared Seedeater *S*. *caerulescens* based on genetic and morphological data, and thus cannot be a female of *S*. *melanops*. We conclude that *Sporophila melanops* is not typical of any natural population of seedeaters, appears to have been collected far from its breeding grounds while overwintering in central Brazil, and should not be afforded any conservation status.

## Introduction

**Fig 1:**
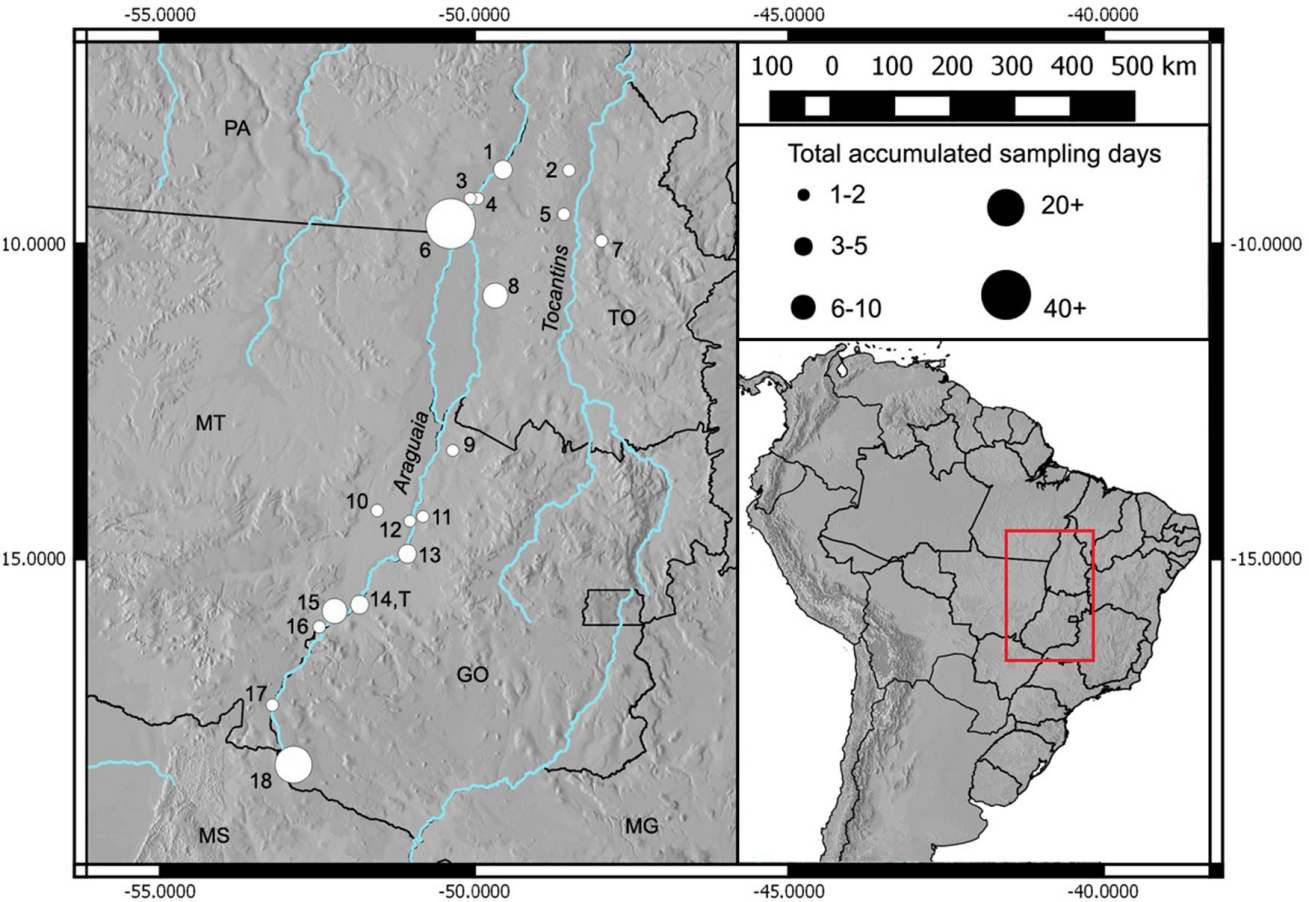
Main localities sampled for *Sporophila* seedeaters in the Araguaia and Tocantins river basins, central Brazil, in 2001–2002 and 2009–2011. The type locality of Hooded Seedeater *Sporophila melanops* is indicated by the letter “T” (= locality 14). Brazilian states are indicated by their official acronyms, as follows: PA = Pará, MT = Mato Grosso, MS = Mato Grosso do Sul, TO = Tocantins, GO = Goiás, and MG = Minas Gerais. Localities: 1. Araguacema; 2. Guaraí; 3. Barreira do Campo; 4. Caseara; 5. Miranorte/Miracema; 6. Fazenda Fartura, Santana do Araguaia; 7. Aparecida do Rio Negro; 8. Lagoa da Confusão 9. Luiz Alves/São Miguel do Araguaia; 10. Pantanal do Rio das Mortes; 11. Road to Rio do Peixe, northern Aruanã; 12. Cocalinho; 13. Aruanã; 14. Registro do Araguaia (type locality); 15. Barra do Garças/Serra Azul; 16. road to Torixoréu; 17. Alto Araguaia/rio Babilônia; and 18. Emas National Park. For a detailed itinerary of the three field trips in 2008–2010 see S3 Appendix.

During his fifth expedition in Brazil, on 19 October 1823, the great Austrian naturalist Johann Natterer collected several seedeaters (*Sporophila* spp.) in the environs of a lake on the east bank of the Rio Araguaia, 3 leagues (*c*. 15 km) north of Porto do Rio Araguaia (= Registro do Araguaia, 15°44’S, 51°50’W; Fig 1), Goiás, in central Brazil. Natterer remained in this area for more than one month (10 October–15 November) before continuing west into Mato Grosso [1]. Forty-eight years later, one of those specimens, a male labeled no. 620 by Natterer, was described by August von Pelzeln as *Spermophila melanops* ([2]: 224, 331–332; S1 Appendix), which was subsequently transferred to the genus *Sporophila*. At the time, Pelzeln was the bird curator at the Naturhistorisches Museum in Wien (NMW) and therefore had all of the *Sporophila* specimens collected by Natterer at hand. To date, this is the only known specimen definitely ascribed to Hooded Seedeater *S*. *melanops*.

Conservation organizations consider the species to be Critically Endangered (Possibly Extinct) [3]. When considering all Critically Endangered species worldwide *S*. *melanops* is that with the longest timespan without records [4], 192 years having elapsed since the type specimen was collected. This has raised suspicion as to its validity as a biological entity and has led to much speculation based on scant data. Most assessments consider *S*. *melanops* to be closely related to Yellow-bellied Seedeater *S*. *nigricollis*, a widespread and common species [5–8]. More recently, the South American Classification Committee has listed *S*. *melanops* as a hybrid or dubious taxon [9], while the Comitê Brasileiro de Registros Ornitológicos accepts it as a valid species until an accurate analysis becomes available [10].

Intriguingly, Meyer de Schauensee [6] examined a female seedeater, also from Goiás, which he considered to be possibly a female *S*. *melanops*. It was collected by G. A. Baer and labeled GB2096 on one of his printed labels. A newer Rothschild Museum label indicates on the reverse side ‘*Sporophila* sp. (*gutturalis*?)’ with a pencil addition indicating ‘*melanops*!’. Identification of female *Sporophila* seedeaters is extremely challenging and sometimes impossible [7,11], leading to doubts concerning the assignation of this female to *S*. *melanops*. Despite its potential importance in solving the riddle posed by *S*. *melanops*, this specimen has not received any previous attention.

Proposed conservation measures for *S*. *melanops* include surveys to search for it in the wild and a re-examination of the type specimen to evaluate the validity of the species [3,12]. Adding to current confusion, most recent illustrations and descriptions have portrayed *Sporophila melanops* as possessing the general colour pattern of *S*. *nigricollis*, including a yellow belly and olive upperparts, differing only in the abrupt interruption of black on the nape and having black restricted to the throat [3, 13–15], sometimes even illustrating a yellow bill. However, another illustration shows *S*. *melanops* as having a black hood, brown back and wings, pale orange belly and flanks, and horn-coloured bill [8].

In this paper we seek to clarify the mystery of *S*. *melanops* by examining all possible information sources, including (a) specific field searches in the wild near the type locality, (b) critical examination of the type specimen and the presumed female, and (c) DNA sequence analysis of both specimens. Based on these novel data, we discuss the conservation, biological and taxonomic status of *Sporophila melanops*.

## Materials and Methods

### Specimen analyses

**Fig 2:**
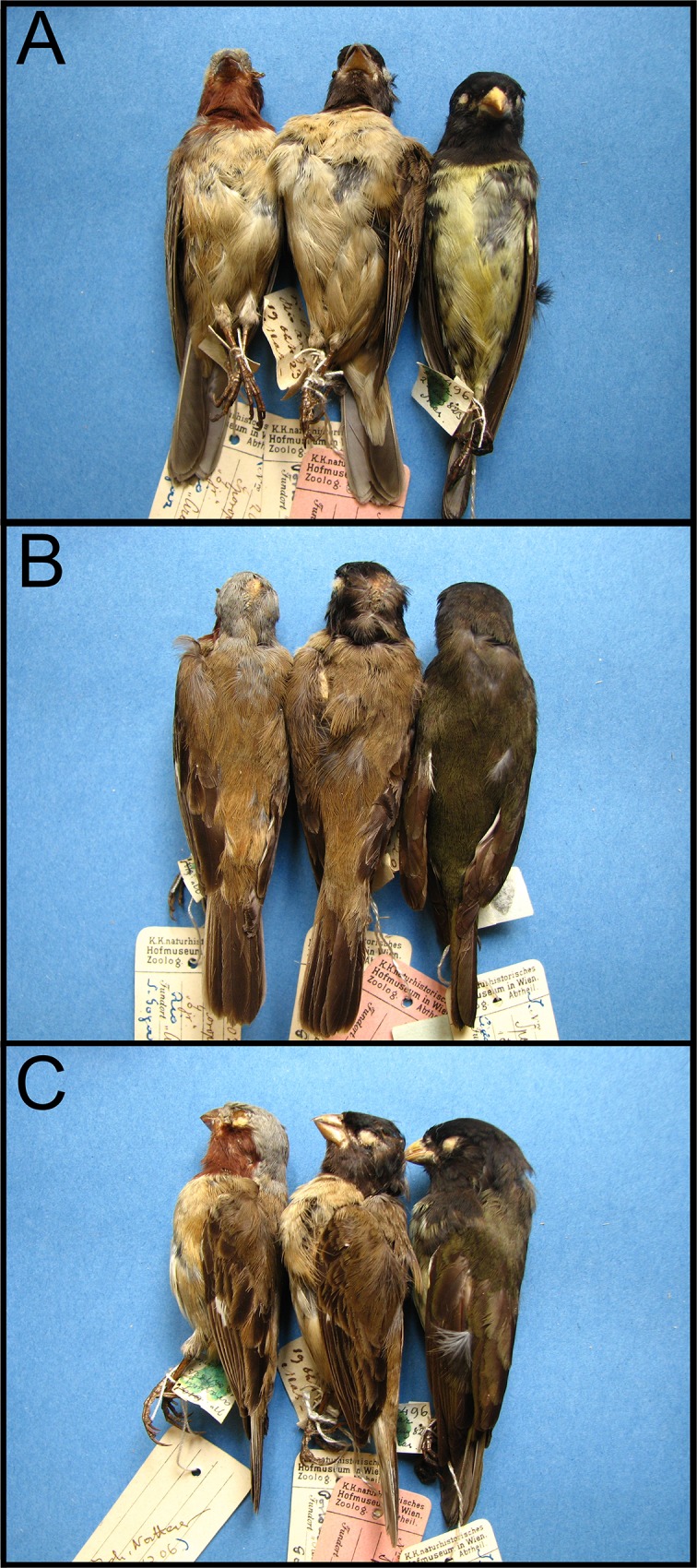
Comparison of the male type specimen of Hooded Seedeater *Sporophila melanops* (centre; NMW 20.316) with an equivalently plumaged male Dark-throated Seedeater *S*. *ruficollis* (left; NMW 20.332) and a fully adult male Yellow-bellied Seedeater *S*. *nigricollis* (right; NMW 20.463). A) Ventral view, B) dorsal view, and C) lateral (left side) view. Throat patch colour and shape, extension and shape of the cap, and dorsal and ventral coloration, as well as mensural data, support the inclusion of *S*. *melanops* within the capuchinos clade. Genetic data strongly support the morphological conclusion.

**Fig 3:**
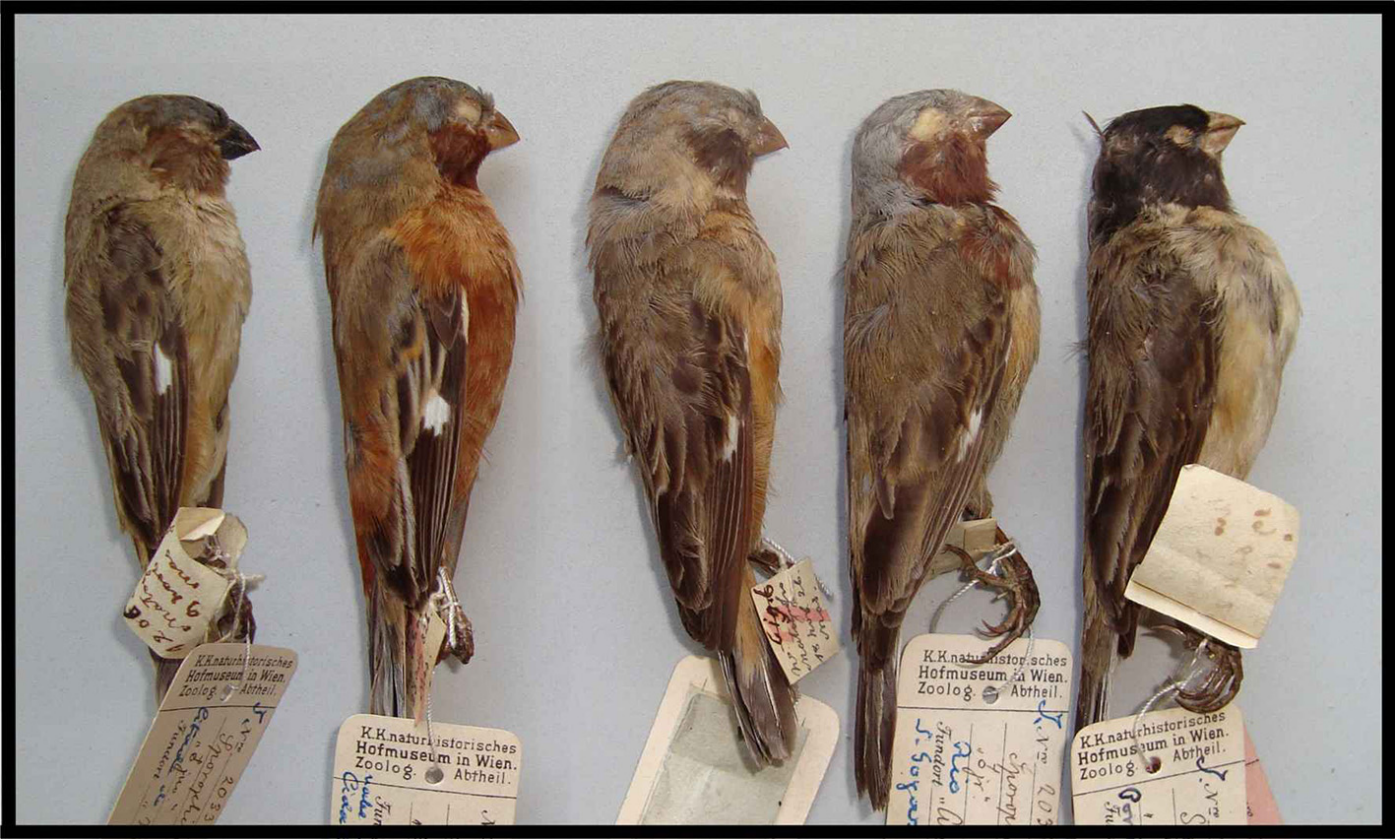
Comparison of the male type specimen of Hooded Seedeater *Sporophila melanops* (first on the right; NMW 20.316) with four differently plumaged male Dark-throated Seedeaters *S*. *ruficollis* (NMW 20.333, 20.330, 20.331, and 20.332) exemplifying intraspecific variation and different stages of breeding plumage acquisition. The specimen to the left of the type was collected at the same time and place, and exhibits the same moult phenology and plumage pattern, further suggesting that the type specimen is an aberrant individual of *S*. *ruficollis* with a black cap.

**Fig 4:**
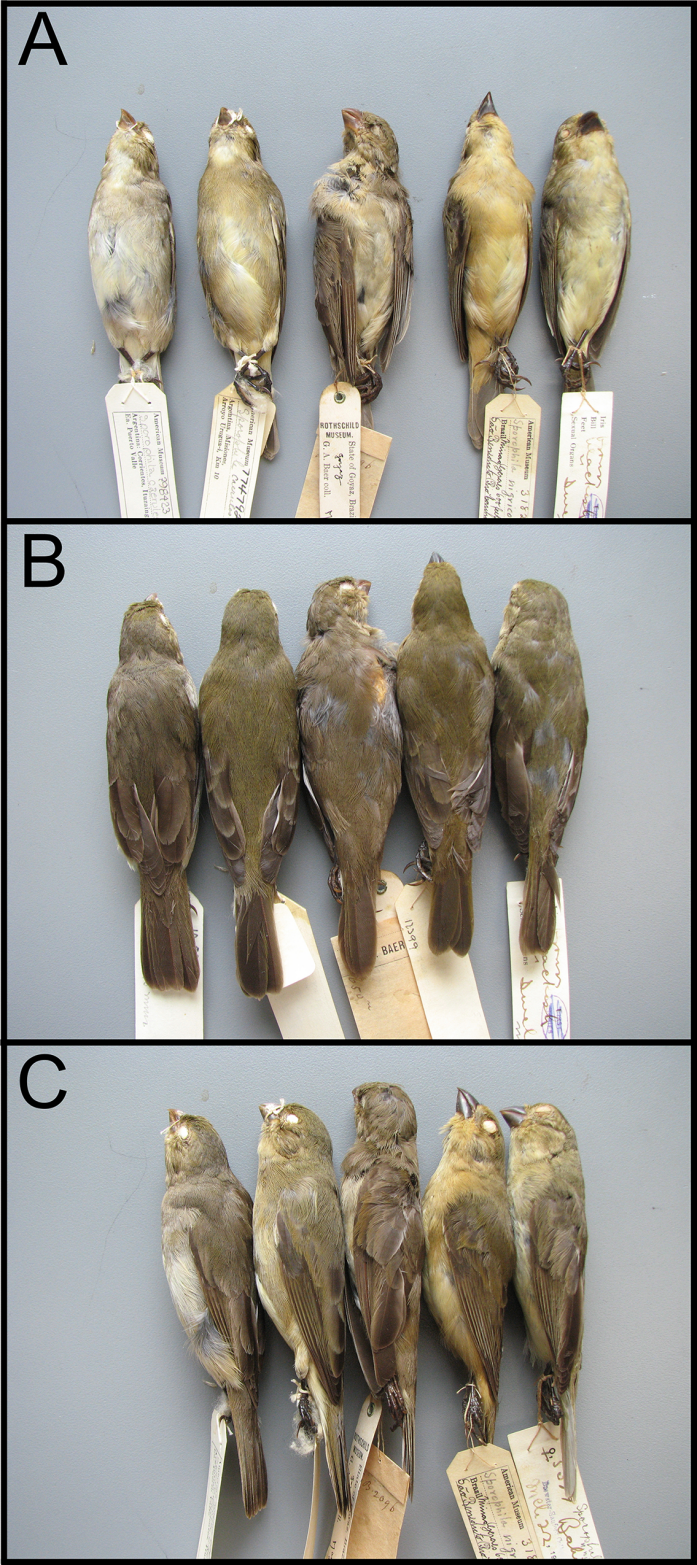
Comparison of the presumed female Hooded Seedeater *Sporophila melanops* (centre; AMNH 514890) with females of Double-collared Seedeater *S*. *caerulescens* (two on the left; AMNH 798423, 774792) and Yellow-bellied Seedeater *S*. *nigricollis* (two on the right; AMNH 318211, 163577). A) Ventral view, B) dorsal view, and C) lateral (left side) view. Morphological and genetic comparisons indicate that this female belongs to the *S*. *caerulescens*/*nigricollis* clade, and not to the capuchinos clade, where both morphological and genetic analyses place the male type of *S*. *melanops*.

The type specimen of *Sporophila melanops* (NMW 20.316, Figs 2 and 3) was carefully examined during two recent, independent visits to the Naturhistorisches Museum in Wien (NMW), Austria, by JIA and VQP. The presumed female specimen of *S*. *melanops* (AMNH 514890, Fig 4) suggested to be such by Meyer de Schauensee [6], was critically examined by JIA at the American Museum of Natural History (AMNH), New York, and directly compared to female specimens of species in all morphological groups of *Sporophila* defined by Ridgely & Tudor [7]. Besides these key specimens, many other specimens of several *Sporophila* species were studied in the two collections cited above and numerous other museums (S2 Appendix).

We measured exposed culmen and tarsus length (to nearest 0.1 mm) and wing chord and tail length (to nearest 0.5 mm) following Baldwin et al. [16]. To increase their comparability with pre-existing measurements, all measurements reported were taken by JIA.

### Genetic analyses

To evaluate the identity of the holotype of *S*. *melanops* and the presumed female based on molecular data, two mitochondrial DNA (mtDNA) marker sequences were sequenced from toe-pad samples: partial sequences of the *cytochrome c oxidase subunit I* (*COI*) and *cytochrome b* (*Cyt-b*) genes (Table 1). Molecular phylogenetic studies have identified nine well-supported clades in *Sporophila* [17]. Given that species in one of these clades (the capuchinos clade) cannot be differentiated based on mtDNA [18] and that we only had fragmentary mtDNA samples stemming from the age of the study material, we compared (a) the genetic divergence of the holotype and the presumed female to all individual samples, and (b) their average genetic divergence to each of the nine well-supported *Sporophila* clades.

**Table 1:** Specimens for which mtDNA sequences were obtained in this study. Sequences of *COI* (+, 176 bp) and of the two different *Cyt-b* fragments: short (s, 176 bp) and long (l, 359 bp), are indicated. Sequences <200 bp are found in S3 Table, those of the long *Cyt-b* fragment are deposited in GenBank (accession numbers KU886564-886565).

Taxon	Sequence name	Voucher specimen, locality, and date of collection	*COI*	C*yt-b*
*S*. *melanops*				
	Spomps1	NMW 20.316 (**holotype of *S*. *melanops* Pelzeln, 1871**), Porto do Rio Araguaia, Goiás, Brazil, 19 Oct 1823	+	s
*Sporophila* sp.				
	Spomps2	AMNH 514890 (**presumed *S*. *melanops* female**), Goiás, Brazil, May 1906	+	s
*S*. *n*. *nigricollis*				
	Sponignig1	NMW 81.607, Limoeiro, Bahia, Brasil, 2 Jan 1981	+	s,l
	Sponignig2	NMW 86.683, Caparo Trinidad, 16 April 1912	+	-
	Sponignig3	NMW 66.327, NW Recife, Brazil, 17 Feb 1903	+	s,l
	Sponignig4	NMW 66.330, Pao d’Alho, Pernambuco, Brazil, 17 Feb 1903	+	-
*S*. *nigricollis vivida*				
	Sponigviv1	NMW 20.454, Paramba, Ecuador, 7 May 1899	+	-
*S*. *lineola*				
	Spolin1	NMW 81.508, Goiania, Goiás, Brasil, 16 May 1980	+	s
*S*. *luctuosa*				
	Spoluc1	NMW 86.693, Yahuaramayo, Puno, Peru, 17 Dec 1912	+	-
*S*. *c*. *caerulescens*				
	Spocaecae1	NMW 80.306, Ituzaingó, Rio Parana, Corrientes, Argentina, 19 Nov 1933	+	s
*S*. *albogularis*				
	Spoalb1	NMW 81.602, Recife, Pernambucco, Brazil, 22 Feb 1971	+	s
*S*. *m*. *minuta*				
	Spominmin1	NMW 86.679, Caparo, Trinidad, 10 Apr 1912	+	-
	Spominmin2	NMW 86.678, Caparo, Trinidad, 10 Apr 1912	+	-
*S*. *hypoxantha*				
	Spohyp1	NMW 80.522, Ituzaingó, Rio Paraná, Argentina, 25 Nov 1983	+	s
*S*. *palustris*				
	Spopal1	NMW 20.339 (**holotype of *S*. *lorenzi* Hellmayr, 1904**), “Cayenne?, Guyana” [*Errore*!], before 1806	+	s
*S*. *castaneiventris*				
	Spocas1	NMW 81.576, Manaus, Brazil, 7 Jun 1965	-	s
*S*. *melanogaster*				
	Spomel1	NMW 20.312 (**syntype**), Ressaca, “Borda do Matto” (= Santo Antonio da Posse), São Paulo, Brazil, 22 Nov 1822	+	s
	Spomel2	NMW 20.311 (**syntype**), Itararé, São Paulo, Brazil, 24 Feb 1821	+	s

Toe-pad samples were used for DNA extraction, which was carried out using the DNA IQ System–Hair and Tissue Extraction Kit (Promega) according to the manufacturer’s instructions. The incubation time was up to 24 hours depending on visible progress of tissue digestion. The final elution volume was 30 μL. Optimum amounts of template DNA were determined empirically (1–10 μL of the DNA solution in 25μl PCR reaction volume). If necessary, re-amplifications were performed with 1–2 μL template DNA. Control extractions without tissue were carried out to detect potential contaminations. To amplify the *COI* sequence the following primers were used: PloCOI-1+ 5´-TTCTCAACCAACCACAAAGA-3´, PloCOI-2- 5´-ATTATCACGAAAGCATGGGC-3´ (size of amplified fragment: 176 bp; sequence without primer sequences: 136 bp). For *Cyt-b* two fragments of different sizes should be amplified: L14841mod 5´-CCATCCAACATCTCAGCATGATGAAA-3´, which was combined either with H15149mod 5´-GCCCCTCAGAATGATATTTGTCCTCA-3´ (size of amplified fragment: 359 bp; sequence without primer sequences: 307 bp) or Plo_cytb1- 5´-AGGTTTCGGATTAGTCAGCC-3´ (176 bp; sequence without primer sequences: 130 bp). PCR was performed on a Mastercycler gradient thermocycler (Eppendorf) in 25 μl containing 2.5 μL of PCR buffer, 3 mM of Mg2+, 0.2 mM of each nucleotide, 0.5 U of Q5 High-Fidelity DNA Polymerase (Biolabs) and 0.5 μM of each primer. PCR profiles comprised an initial heating step at 94°C for 3 min followed by 45 cycles: 30 s at 94°C, 30 s at annealing temperature (54°C for Cyt-b and 63°C for COI) and 60 s at 72°C. After the last cycle, a final extension of 7 min at 72°C was performed. Control PCR reactions to detect potential contaminations were carried out with: (i) control DNA extractions, and (ii) with distilled water instead of the template. PCR products were purified using the QIAquick PCR Purification Kit (Qiagen Inc.) and sequenced directly in both directions (using the amplification primers) at LGC Genomics (Berlin, Germany). Editing of sequences was performed using BioEdit v. 7.0.1 [19]. To confirm the validity of the PCR results, samples of crucial specimens were repeatedly amplified, each repetition starting with new DNA extractions (holotype of *S*. *melanops*: *Cyt-b* four times, *COI* two times; presumed female: *Cyt-b* two times). To evaluate the predictive power of the sequences obtained from old specimens, we sequenced 16 additional specimens (10 species) whose identity is undisputed and performed the same analyses made for the holotype and the presumed female. All individuals sequenced in the present study were adult males except the presumed female of *S*. *melanops* and a specimen of Tawny-bellied Seedeater *S*. *hypoxantha*, which is an adult female (Table 1).

We obtained all sequences of *Sporophila* (including *Dolospingus* and *Oryzoborus*) available in GenBank for *Cyt-b* and *COI* genes and aligned them with our sequences using the software MEGA6 [20]. We inspected the alignment visually and discarded the sequences with less than 85 bp of overlap with our fragments. GenBank sequences of a species belonging to one clade but with a complete match (i.e., 0% divergence) to a species belonging to a different clade were also discarded. Our final dataset included 155 (*Cyt-b*) and 165 (*COI*) sequences belonging to 32 of the 39 *Sporophila* species and representatives of all main lineages [17], plus *S*. *melanops* (S1 and S2 Tables). We calculated the genetic divergence (*p*-distance) of the male holotype of *S*. *melanops* and the presumed female to all sequences of each marker with pairwise deletion of missing data in MEGA6 [20]. Samples of *Sporophila americana* were assigned to Clade V based on Stiles [21] and that of the bamboo-specialist *S*. *frontalis* was treated as a distinct lineage due to its uncertain phylogenetic position [22].

### Fieldwork

Because of the surprising lack of specific searches for *S*. *melanops* by modern ornithologists, we made several expeditions to the region of the type locality and neighbouring areas of the Araguaia Valley, as well as north into the neighbouring states of Pará and Tocantins, specifically searching for *S*. *melanops* (Fig 1). These searches were undertaken in December 2008 / January 2009 (the local breeding season), October / November 2009 (at the type locality seven days after the corresponding date when the type was collected), and July 2010 (when many austral migrants might have been expected to be present). A detailed itinerary of the localities visited is presented within the Supplementary Information (S3 Appendix).

The usual approach was to search for suitable marshy wetlands with high likelihood for finding seedeaters, attempting to cover as much of the region as possible, but stopping wherever we found such habitat. We questioned local people about the presence of potentially suitable areas for *Sporophila*, to aid our searches and save time.

Data from two trips to Emas National Park between 4–16 October 2001 and 30 September–6 October 2002 by VQP are also included, although neither trip focused specifically on searching for *S*. *melanops*. Altogether, our effort searching for *Sporophila* seedeaters in the Araguaia River basin and nearby localities totals *c*. 100 days.

## Results

### The male type specimen of *S*. *melanops*

A number of descriptions of this specimen (NMW 20.316, Figs 2 and 3) taken from the literature have been compiled to facilitate comparison (S1 Appendix). Our detailed examination is generally consistent with the descriptions provided by Pelzeln [2] and Hellmayr [23–24]. However, we noticed two important features that had previously gone undetected. First, the hood of *S*. *melanops* is not uniformly black. Instead, the cap and nape are pure black, whereas the cheeks and throat have a reddish, dark coffee colour only visible at certain angles (Figs 2 and 3). Second, there are a few dark feathers, largely concolorous with the throat (albeit perhaps marginally paler), on the right side of the breast and lower flanks, and a few olivaceous feathers on the nape.

The descriptions of Pelzeln and Hellmayr differed in two respects. First, Pelzeln [2] described the white half eye-ring below the eye (more visible on the left side according to our examination; Fig 2), but this was omitted by subsequent authors (except Sick [25]). Second, Hellmayr [23–24] described the bill as being “stouter, shorter and with a more rounded culmen” whereas Pelzeln [2] did not make any special comment concerning this feature.

The *S*. *melanops* type is certainly an adult based on the shape of remiges and rectrices, both of which are much less pointed than in juveniles [26]. The specimen measured: exposed culmen 8.1 mm, wing length 54.5 mm, tail length 38.5 mm and tarsus length 14.5 mm. In comparison, ten males of *S*. *nigricollis* measured: exposed culmen 8.45 ± 0.20 (8.21–8.93) mm, wing length 55.05 ± 1.95 (52.50–57.50) mm, tail length 43.10 ± 1.17 (42.00–45.00) mm, and tarsus length 14.44 ± 0.50 (13.73–15.31) mm.

### The putative female of *S*. *melanops*

There is no critical description or evaluation of this specimen (AMNH 514890, Fig 4) in the literature, other than its original mention ([6]; see S1 Appendix for Meyer de Schauensee’s description). Compared to other female seedeaters, it bears more resemblance to females of *S*. *nigricollis* than to those of any other species. It is in heavy moult of the remiges and body feathers, and it lacks any speculum or wing-band. The dorsal colour is not as greenish as in typical female *S*. *nigricollis*, being somewhat intermediate between the bright olive-green of *S*. *nigricollis* and reddish brown of Plumbeous Seedeater *S*. *plumbea*. However, the dorsal colour of some female *S*. *nigricollis* approximates closely to this specimen. In this female, more typical greenish-olive (recently replaced?) feathers are intermingled with greyer ones (worn?) especially on the forehead and above the eye. Bill shape and size fall within the variation of *S*. *nigricollis*. The ill-defined brownish colour of the tail feathers, which have greenish fringes, fits perfectly well the known variation within *S*. *nigricollis*. The bill is yellowish with darker areas, a pattern found regularly in wintering *Sporophila*. The supposed ventral colour difference between the presumed female *S*. *melanops* and *S*. *nigricollis* ([6], S1 Appendix) falls within the variation found in *S*. *nigricollis*, and more frequently in *S*. *caerulescens* (which has a whiter belly centre and less yellowish around it). Upper wing-coverts have pale ferruginous-brown fringes, differing from the more greenish fringes of *S*. *nigricollis*, and resembling the condition found in many young *Sporophila*. At first sight, the bill appears bulkier (more voluminous) than that of most *S*. *nigricollis* (perhaps because the maxilla and mandible are perfectly aligned, with no protrusion of the maxilla), but it is smaller than that of *S*. *plumbea* (no size overlap) and within the variation shown by *S*. *nigricollis*.

The specimen measured: exposed culmen 8.25 mm, wing chord 56 mm, tail length 45 mm, and tarsus length 14.61 mm.

### Genetic analysis of the male type and of the putative female of *S*. *melanops*

**Fig 5:**
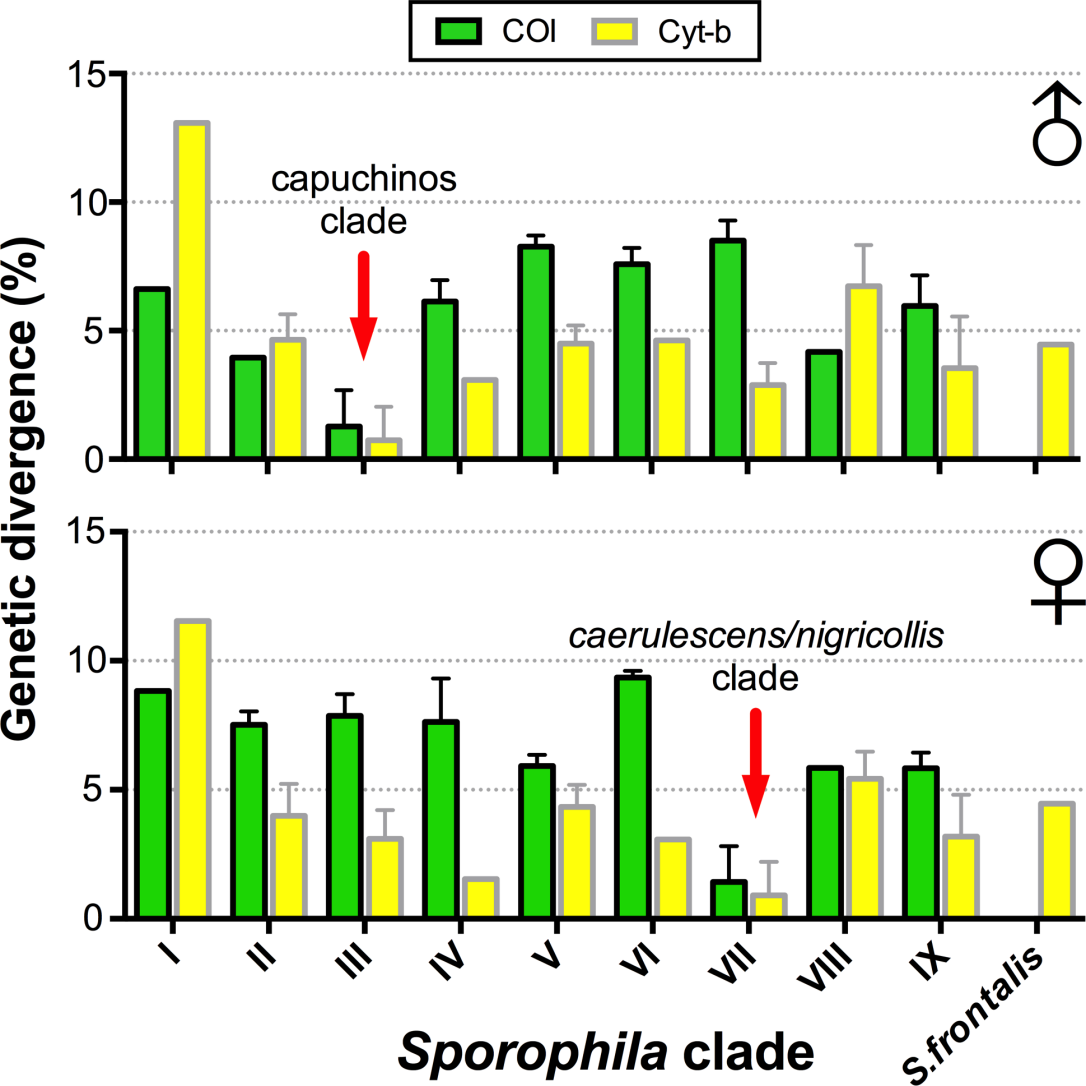
Mean (±SD) genetic divergence (*p*-distance) of the male holotype of Hooded Seedeater *Sporophila melanops* (above) and the presumed female (below) to samples from the nine well-supported clades of *Sporophila* [17] and *S*. *frontalis* based on sequences of *COI* and *Cyt-b* genes. The red arrows indicate the clade to which each specimen belongs based on the lowest average distances in both genes and given the presence of many identical gene sequences in the same clade. See also S1 and S2 Tables.

Average and minimum genetic divergences of the male holotype of *S*. *melanops* to other *Sporophila* clearly show that it belongs to the capuchinos clade (Fig 5; clade III). The *COI* sequence of the holotype is identical to that of 18 individuals (five species) in the capuchinos clade (all from the “southern capuchinos” group [18]) and differs by 3.94–10.29% from all samples belonging to the remaining clades (60 individuals). Its *Cyt-b* sequence is identical to that of 70 individuals (seven species) in the capuchinos clade and differs by 1.59–13.08% from the sequences in the other clades (52 individuals) (Fig 5 and S1 and S2 Tables). Mean and minimum genetic divergences of the presumed female of *S*. *melanops* to other *Sporophila* indicate that it belongs to the *caerulescens*/*nigricollis* clade (Fig 5; clade VII). The *COI* sequence of the female specimen is identical to that of nine *S*. *caerulescens* and one *S*. *nigricollis* and differs by 5.15–11.65% from those in other clades (141 individuals). Its *Cyt-b* sequence is identical to that of seven *S*. *caerulescens* and four *S*. *nigricollis*, and differs between 1.54 and 11.54% from all samples of the other clades (138 individuals; Fig 5 and S1 and S2 Tables). The holotype and the presumed female differ by 3.08% and 8.82% in their *Cyt-b* and *COI* sequences. Thus, the genetic data are in complete agreement with the morphological data in demonstrating that the holotype belongs to the capuchinos clade and the female specimen to the *S*. *caerulescens*/*nigricollis* clade.

All our newly sequenced samples of specimens with known identity were most similar, usually identical to, samples from the clade to which they were expected to belong (S1 and S2 Tables). This indicates that our short mtDNA fragments have the power to confidently place the holotype of *S*. *melanops* and presumed female at clade level. We also sequenced the type specimen of *S*. *lorenzi*, a species described by Hellmayr [23] but which was subsequently considered to be an artefact, with a body pertaining to *S*. *palustris* and wings from an unknown species [24]. Our examination of the type agrees with Hellmayr’s [24] evaluation, and the placement of *S*. *lorenzi* in the capuchinos based on the molecular data is also as expected (S1 and S2 Tables).

### Fieldwork

No birds showing the plumage features of *S*. *melanops* were observed during the fieldwork (S3 Appendix). However, these efforts were of sufficient magnitude to find all species of *Sporophila* seedeaters expected to occur in the Araguaia basin. The following species of seedeaters were encountered during our surveys (number of days on which the species was recorded in parentheses):

Trip 1 (December 2008–January 2009): Plumbeous Seedeater *Sporophila plumbea* (10), Rusty-collared Seedeater *S*. *collaris* (6), Lined Seedeater *S*. *lineola* (6), Yellow-bellied Seedeater *S*. *nigricollis* (8), White-bellied Seedeater *S*. *leucoptera* (7), Copper Seedeater *S*. *bouvreuil* (1), Pearly-bellied Seedeater *S*. *pileata* (6), Tawny-bellied Seedeater *S*. *hypoxantha* (1, only in Emas National Park), Chestnut-bellied Seed Finch *S*. *angolensis* (8) and Great-billed Seed Finch *S*. *maximiliani* (2, only in Emas National Park and at Caseara, Tocantins). Virtually all of these observations were made in marshy or wet areas abutting gallery forests, or in wet grassy *brejos*, except at Aruanã, where the birds were found in grassy vegetation and dense stands of bamboo on dry land.

Trip 2 (October–November 2009): *Sporophila plumbea* (1), *S*. *collaris* (6), *S*. *lineola* (1), *S*. *nigricollis* (2), *S*. *caerulescens* (1), *S*. *leucoptera* (1), *S*. *bouvreuil* (7), Chestnut Seedeater *S*. *cinnamomea* (1) and *S*. *angolensis* (3).

Trip 3 (July 2010): *Sporophila plumbea* (5), *S*. *collaris* (7), S. *nigricollis* (6), *S*. *caerulescens* (4), *S*. *leucoptera* (4), *S*. *bouvreuil* (4), Marsh Seedeater *S*. *palustris* (2), *S*. *cinnamomea* (2), *S*. *hypochroma* (2), *S*. *hypoxantha* (2), *S*. *ruficollis* (2) and *S*. *angolensis* (3).

Visits to Emas National Park (October 2001; October 2002): *Sporophila plumbea*, *S*. *caerulescens*, *S*. *pileata* and *S*. *palustris*, usually in the marshy grassland on the banks of the Rio Formoso (October 2001). These same species, plus *S*. *cinnamomea* and *S*. *hypoxantha*, were found in a single mixed-species flock at the same site (October 2002).

Additionally, five two-week surveys of birds at Fazenda Fartura, Santana do Araguaia, resulted in records of *S*. *plumbea*, *S*. *collaris*, *S*. *lineola*, *S*. *nigricollis*, *S*. *leucoptera*, *S*. *bouvreuil*, *S*. *palustris*, *S*. *hypoxantha*, *S*. *ruficollis* and *S*. *angolensis* (results summarized in [27]). Despite observing huge flocks of seedeaters, sometimes with more than 1000 birds, none with a plumage similar to the type specimen of *S*. *melanops* was seen [28]. Likewise, other recent surveys of *Sporophila* seedeaters in the region failed to record any *S*. *melanops* [29].

## Discussion

Morphological and genetic assessments of the male type of *S*. *melanops* clearly show that it belongs to the capuchinos clade. The capuchinos represent an extremely complex group with a controversial taxonomic history resultant, in part, from an apparently recent radiation in which scant genetic differentiation has nonetheless produced a vast array of plumage, ecological and vocal variation [11, 30–33]. Concordant with this, there is no indication of genetic differentiation between the capuchinos in our sample (*S*. *melanops*, *S*. *melanogaster*, *S*. *hypoxantha* and *S*. *palustris*, the latter including *S*. *lorenzi*). As our genetic results are based on very short sequences, it must be emphasized that the distances may not be directly compared with distances obtained from complete genes. Our general results are consistent with all recent molecular phylogenies of *Sporophila* [17–18, 33–36], and more importantly, placement of the male type of *S*. *melanops* and of the presumed female were unequivocal at the clade level according to both sampled genes.

The plumage pattern, measurements and phylogenetic position of the type of *S*. *melanops* strongly suggest that it is an adult capuchino moulting from non-breeding to breeding plumage with an exceptional melanistic crown. The overall plumage exhibited by the type of *S*. *melanops* is precisely like that found in several capuchino males in nature: a fully-coloured hood with a young/female-like body. We have repeatedly observed this pattern in the wild and it is thought to occur in both young males acquiring adult plumage and adult males regaining breeding plumage. It can be seen in museum specimens and in photographs of live birds (e.g., *S*. *ruficollis* NMW 20.332, 20.333, WA 469585, *S*. *palustris* MZUSP 84102; WA 768500, *S*. *cinnamomea* WA 17057, WA 818319, and *S*. *melanogaster* WA 225539, WA 226122; WA refers to photos at www.wikiaves.com.br/[photo number]). Measurements (especially exposed culmen and tail length) agree with those of species in the capuchinos group [11, 30, 32]. However, the combination of a dark, brownish throat and upper breast, with a pure black crown and nape is unique among capuchinos or any other *Sporophila* species.

The presumed female *S*. *melanops* [6] is not closely related to the male type, and instead belongs to the *S*. *caerulescens*/*nigricollis* clade based on morphological and genetic data. Although we cannot confirm that this female is a *S*. *nigricollis* based solely on morphological evidence, all available information is consistent with this idea. On the other hand, measurements and plumage features reject the possibilities that it (1) is a female *S*. *melanops* and (2) is a female capuchino.

### Previous hypotheses for *S*. *melanops*

A cornucopia of treatments is available for *S*. *melanops*, encompassing virtually all possibilities. Sclater ([5]: 19), who never saw the type specimen, thought *S*. *melanops* was a valid species closer to Double-collared Seedeater *S*. *caerulescens* (perhaps stemming from his confusion between *S*. *nigricollis* and *S*. *caerulescens*, for which see Hellmayr [24]: 205 and Sclater [5]: 12–13). Hellmayr [23–24] examined the type specimen and considered it a very distinct species, but noted its superficial resemblance to *S*. *nigricollis*. Thereafter, all taxonomic treatments and proposals relied on published descriptions of the type without personally examining it. Bertoni [37] suggested that it be included within the “Pico Grueso Variable” of Azara [38]. Meyer de Schauensee [6] considered it a valid species allied to *S*. *nigricollis*, but later included it among birds of doubtful validity [39]. Paynter and Storer [40] listed it as a valid species. Ridgely and Tudor [7] doubted its validity, and despite differences in plumage pattern, suggested that it seemed closely related to *S*. *nigricollis*, perhaps an aberrant individual, but without discarding the potential for a hybrid origin. These views formed the basis for subsequent treatments (e.g., Sibley and Monroe [41]). Although maintained by Collar et al. [12] and in all subsequent BirdLife International [3] reviews of globally threatened birds, *S*. *melanops* was not listed by Stotz et al. [42] or Gill and Wright [43]. On the other hand, Sick [25] mentioned it quite uncritically, whilst presenting what he considered to be its distinguishing characters, and Dickinson [44] also considered it a valid species without commenting on its taxonomic status. Rising and Jaramillo [8] provisionally included it within *S*. *nigricollis*.

Four facts strongly suggest that *S*. *melanops* was an overwintering bird ready to return to its breeding grounds further south, and not a breeder in the Araguaia River basin as traditionally thought: (1) the date of collection of *S*. *melanops* (October, when migrants from the south are still overwintering in central Brazil), (2) the context in which the specimen was collected (a mixed-species flock of capuchinos including other southern breeders as winter visitors to the region including *S*. *ruficollis*, *S*. *bouvreuil* and *S*. *cinnamomea*), (3) the yellowish bill colour and its ill-defined pattern (typical of birds in non-breeding plumage which frequently occur in mixed-species flocks of overwintering capuchinos [45–46]), and (4) the heavy moult of the specimen (which is expected to occur before migration and not at the breeding grounds before the onset of the breeding season).

### What *S*. *melanops* is not

The phylogenetic position of *S*. *melanops* is inconsistent with it being an aberrant *S*. *nigricollis* as widely held. Moreover, three morphological features of the male *S*. *melanops* clearly separate it from *S*. *nigricollis*. First, it has pale-creamy underparts and brownish dorsal plumage, thus lacking the greenish tinge to the upperparts that is a constant character in all developmental stages between young and adult in *S*. *nigricollis*. Second, it has a bicoloured hood, unlike the black hood of *S*. *nigricollis*. Third, it displays a sub-ocular white crescent typical of the capuchinos that is never present in *S*. *nigricollis*. Even if one accepts that the controversial Dubois’s Seedeater *S*. *ardesiaca* is a morph of *S*. *nigricollis*, differing from the latter in its white belly and grey back, *S*. *melanops* can be separated by the presence of the last two characters. Additionally, measurements of the holotype of *S*. *melanops* (especially exposed culmen and tail length) are shorter than the lowest values for *S*. *nigricollis*. In sum, we can reject the hypothesis that *S*. *melanops* is a young, variant or aberrant individual of *S*. *nigricollis* [7–8].

For *Sporophila melanops* to be a valid biological or phylogenetic species, its type specimen should be a representative member of a natural population of seedeaters distinct from its congeners. Our failure to find *S*. *melanops* despite our intensive searches in and around the type locality, in adequate habitat, and at different seasons of the year, plus surveys in most of the suitable habitats for capuchinos in Brazil, Argentina, Paraguay, Uruguay and Bolivia over 10+ years, strongly suggest there is no such population (for an approximation of our additional sampling effort see [31, 46]). This conclusion is reinforced by the absence of any record (specimen, photograph or sighting) of a *Sporophila* resembling *S*. *melanops* since the collection of the type in 1823. Undoubtedly valid species in the capuchinos clade, to which *S*. *melanops* belongs, are quite habitat-specific and are common in their preferred habitat, while scarce plumage types have been demonstrated to be morphs or, less likely, hybrids [11,30,32]. It is evident, based on the single male and from the failure of our surveys to find *S*. *melanops*, that it does not share these properties with undoubtedly valid species in the capuchinos clade. Thus, we reject the hypothesis that the male type *S*. *melanops* is a typical representative of a species in the capuchinos clade on its own.

The *ad-hoc* hypothesis that the type of *S*. *melanops* represents one of the last specimens of a now-extinct, range-restricted species can be put forward. However, (1) there is no evidence of other extinctions of birds known to breed in the same region, (2) it seems likely that the male *S*. *melanops* was wintering in the area and not a breeder, and (3) other long- and mid-distance migratory *Sporophila* still occur regularly in the area, suggesting that habitat modifications have not altered their normal phenology. We consider that there is no evidence to recognize *S*. *melanops* as a representative of a good species. However, caution is advised on taking this conclusion too far, as discussed next.

If *S*. *melanops* is a hybrid (an improbable hypothesis), the hybridization event probably occurred well away from the type locality and between two capuchinos. However, we lack sufficient evidence to permit a critical evaluation of a hybrid origin for *S*. *melanops*, since we only analysed female-inherited mtDNA, which shows that at least the female parent of the male *S*. *melanops* was a capuchino. However, a hybridization hypothesis will need to take into consideration that, on morphological grounds, the most likely sources of hybridization would be: (1) a dark-capped capuchino on one side (namely, *S*. *bouvreuil*, *S*. *pileata* or *S*. *nigrorufa*), and (2) a colour-throated capuchino on the other (namely, *S*. *ruficollis*, and less likely *S*. *hypochroma*). However, the range of combinations in nature is restricted by the geographic distribution of potential parental species: only *S*. *pileata* coexists spatially with *S*. *ruficollis* and with *S*. *hypochroma* [7,47]. The fact that the southern capuchinos are not differentiated using microsatellite markers [33] or Single Nucleotide Polymorphisms [36] implies that a proof of hybridization based on molecular genetic markers is unfeasible. A definite test of this hypothesis appears to be, at present, impossible.

### What *S*. *melanops* is

Oddly-plumaged *Sporophila* seedeaters are not uncommon, as exemplified by several rare morphs of the capuchinos [11,30,32], and *S*. *melanops* probably represents another example of such phenomena. If *S*. *melanops* is an oddly-plumaged capuchino, to which species does it belong?

The bird number 126, Pico Grueso Variable, of Azara [38] has been shown to include various species of capuchinos [30]. Bertoni [37, 48] proposed that *S*. *melanops* belonged to this composite species. However, none of the multiple putative male plumages described by Azara fully corresponds with *S*. *melanops* as detailed here.

Other capuchinos are *prima facie* good candidates as sources for *S*. *melanops*. Both *S*. *bouvreuil* and *S*. *pileata* have only a black cap, which differs from the black cap extending down to the nape and sharply marked around the ear-coverts of the type *S*. *melanops*. The type shares the black cap and nape with *S*. *nigrorufa*, but the possibility that the type is an oddly dark-throated *S*. *nigrorufa* is very unlikely, as this range-restricted species found in western Brazil and Bolivia does not migrate to the Araguaia River basin or elsewhere in central Brazil.

Our favoured candidate is *S*. *ruficollis*. Some individuals share the dark sepia throat with the type of *S*. *melanops*, and it is a common wintering capuchino in the Araguaia Valley that was moreover collected together with the type. In fact, such a specimen of *S*. *ruficollis* matches very well the moulting phenology and plumage pattern of the *S*. *melanops* type (i.e. head with breeding plumage, body mostly with female-like plumage and a few dark/coloured feathers on the right side of the breast; Fig 3, specimen NMW 20.332 immediately to the left of the *S*. *melanops* type). In contrast, the two additional species collected by Natterer at the type locality (*S*. *cinnamomea* and *S*. *bouvreuil*) were almost completely adult/breeding-plumaged birds (e.g., *S*. *bouvreuil* NMW 20.349, and *S*. *cinnamomea* NMW 20.313–20.315), therefore displaying a completely different moulting phenology. In summary, it is very likely that *S*. *melanops* is an oddly black-capped *S*. *ruficollis*: matching the type to any other *Sporophila* species would require several additional odd events (e.g., *S*. *hypoxantha*, besides not having been collected with the type, would require an oddly-coloured cap, and an oddly-coloured throat). We speculate that *S*. *melanops* in full breeding dress might look like a relatively dark-throated male *S*. *ruficollis* but with a black cap, nape and back. However, given the large amount of plumage variation exhibited by capuchinos and uncertainties in the taxonomy of the *S*. *ruficollis* group [11,30,47,49] this remains an informed suggestion.

One intriguing possibility remains to be tested. A local population of *Sporophila* seedeaters with diagnostic vocalizations was first sound-recorded by JIA in November 2005 at the Esteros del Iberá in Estancia Rincón del Socorro, Corrientes, Argentina, and genetic samples were obtained by Mark Pearman in the late 1990s, with several birds recently photographed at other localities. It exhibits a grey cap, narrow dark throat variably extending to the sides of neck or the nape, pale creamy belly with occasional tawny markings and brownish upperparts, and is very similar or indistinguishable from some plumages of the ‘caraguata’ form described by Areta et al. [30]. The form from the Esteros del Iberá could represent the normal phenotype of the population to which the aberrantly capped *S*. *melanops* type could have belonged. Testing this hypothesis is extremely difficult, but this possibility should be seriously considered given the overall similarity of these forms. Deciding whether what is actually known as *S*. *ruficollis* must be known as *S*. *plumbeiceps* [30], while the name *S*. *ruficollis* would become available for the apparently distinct population of seedeaters in Esteros del Iberá is tightly linked to elucidation of the validity of the name *S*. *melanops*, which might prove to be a junior synonym of *Sporophila ruficollis* Cabanis, 1851, or a senior synonym of *Spermophila plumbeiceps* Salvadori, 1895. These issues must be resolved before deciding whether the Esteros del Iberá birds deserve description as a new taxon [50, 51].

Finally, an especially relevant case of an abnormal capuchino may shed light on the identity of *S*. *melanops*. A chestnut-plumaged adult male capuchino with a black crown was photographed in the Esteros del Iberá, Corrientes, Argentina, by A. Parera. It was thought to be either a male *S*. *cinnamomea* with melanism in the cap (JIA), an extremely dark male *S*. *bouvreuil* outside its regular distribution (EM), or less likely a hybrid between *S*. *cinnamomea* x *S*. *pileata* (JIA). This case is similar to that of *S*. *melanops* in that an otherwise normally plumaged male displayed a black cap instead of a grey one.

### Conclusions

We conclude that there is no evidence to support *S*. *melanops* as a typical representative of a good species or some form of *S*. *nigricollis*, as historically contended, and that its true affinities lie with the capuchinos based on plumage pattern, morphology and mtDNA. Within this group, a hybrid origin seems improbable but very difficult to test. Our analyses suggest that *S*. *melanops* is an aberrant male *S*. *ruficollis* with a melanistic cap acquiring breeding plumage. Collection date, context, bill morphology and moult of the *S*. *melanops* type further indicate that it was not a local breeder in the Araguaia River basin, but an overwintering migrant from the south. The presumed *S*. *melanops* female can be correctly identified as belonging to a different clade of *Sporophila* seedeaters including *S*. *nigricollis* and *S*. *caerulescens* based on morphological and genetic data. Our analyses of the type, further fostered by recent advances in our understanding of the plumage variation and systematics of the capuchinos seedeaters, allowed us to reach a completely novel conclusion with respect to the phylogenetic placement and validity of *S*. *melanops*.

The fact that there appears to never have been a population of birds with the appearance of the until-now Critically Endangered (Possibly Extinct) *Sporophila melanops* should not reduce the conservation importance of its presumed habitat in central Brazil. The preservation of natural grasslands of the Araguaia and Tocantins river basins, as well as several other similar areas in São Paulo, Minas Gerais and Bahia states, is important to assure the conservation of the breeding and wintering grounds of many threatened species of *Sporophila* seedeaters. Although Critically Endangered *Sporophila* species have been “lost” following careful reviews of their validity, elucidating the identity of questionable species such as *S*. *insulata* [52], *S*. *zelichi* [11], and now *S*. *melanops* (this work) permits conservationists to realistically focus resources on those species that are truly threatened.

## Supporting Information

S1 Appendix Specimen descriptions. Selected plumage descriptions and literal transcriptions of detailed taxonomic considerations in old references.
(DOCX)

S2 Appendix Museum specimens examined. Most relevant specimens examined for this study. See also other examined specimens in Table 1 and in our previous publications [11,30–32,38,47,49].
(DOCX)

S3 Appendix Field searches. Detailed itinerary of the field trips searching for *Sporophila melanops* in the Araguaia River basin.
(DOCX)

S1 Table Genetic *p*-distances for *COI*. Pairwise divergence of the sequences of the male holotype of *Sporophila melanops* and the presumed female to available sequences of *Sporophila* in GenBank (as of 29 February 2016). Roman numerals refer to the well-supported clades identified by Mason and Burns [17].
(DOCX)

S2 Table Genetic *p*-distances for *Cyt-b*. Pairwise divergence of the sequences of the male holotype of *Sporophila melanops* and the presumed female to available sequences of *Sporophila* in GenBank (as of 29 February 2016). Roman numerals refer to the well-supported clades identified by Mason and Burns [17].
(DOCX)

S3 Table Genetic sequences. Short fragments (<200 bp) of *COI* and *Cyt-b* genes of specimens sequenced for the present study.
(DOCX)
