# Supplementary material for: Tiny Bird, Huge Mystery—The Possibly Extinct Hooded Seedeater (Sporophila melanops) Is a Capuchino with a Melanistic Cap
Source: PLoS One. 2016 May 11;11(5):e0154231. doi: 10.1371/journal.pone.0154231 (PMC4864415; doi:10.1371/journal.pone.0154231)
Supplement: S1 Appendix — Selected plumage descriptions and literal transcriptions of detailed taxonomic considerations in old references. (DOCX) [file pone.0154231.s001.docx]

**PLOS One**

**Tiny bird, huge mystery—the Possibly Extinct Hooded Seedeater (*Sporophila melanops*) is a capuchino with a melanistic cap**

Juan Ignacio Areta, Vítor de Q. Piacentini, Elisabeth Haring, Anita Gamauf, Luís Fábio Silveira, Erika Machado, Guy M. Kirwan

**S1 Appendix. Specimen descriptions.** Selected plumage descriptions and literal transcriptions of detailed taxonomic considerations in old references.

**A)** **Original description of *Sporophila melanops* Pelzeln.**

Pelzeln (1870: 331-332; see also page 224 where the name is first used):

**“Spermophila melanops** Natterer. No. 620.

Sp. capite, nucha, regione auriculari et gula circumscripte nigris, notæo flavescente cinereofusco, gastræo brunnescente albo hypochondriis ochraceo lavatis, remigibus 1. 2. 3. 8. 9. Sine albedine, 4. 5. 6. 7. Basi utriusque pogonii, 10. 11. 12. 13. 14. Basi pogonii interni albis. Longit. 4 ½ “, alae 2” 1”’, caudae 1” 7”’, rostri a rictu 4”’, tars. 6½”’.

Loxia melanops Natterer Catal. Msc.

Hab. Porto do Rio Araguay.

Ad *Sp. ornatam* (Licht) proxima accedit.

Natterer’s Notizen: Männchen (stark in der Mauser, Porto Rio Araguay an einer lagoa drei Meilen nördlich in Gesellschaft mit anderen Gimpeln, Oktober). Iris dunkelbraun, Oberschnabel und Spitze des Unterschnabels gelbbraun, der übrige Schnabel bräunlichgelb. Länge 5 ¼”, breite 7”, der Schwanz ragt 12 ½”’über die Flügelspitzen.

Ganzer Kopf, Gurgel, Ohrengegend und Nacken rund abgeschnitten, glänzend Schwarz, der übrige Oberleib hell gelblich graubraun, Unterseite bräunlichweiss an den Seiten in Ochergelb übergehend, Flügel und Schwanzfedern dunkelbraun, die 1. 2. 3. ohne Weiss, die 4. 5. 6. 7. Schwungfeder mit weisser Wurzel an beiden Fahnen, die 8. 9. ganz ohne Weiss, die 10. 11. 12. 13. 14. mit weisser Wurzel an der inneren Fahne. Die oberen Flügeldeckfedern schwarzbraun mit hell graubraunen Rändern, die Schwung und Schwanzfedern ebenso gerändert, die Federchen an der unteren Hälfte der Augenringe weiss.”

**Translation:** “**Spermophila melanops** Natterer. No. 620.

Sp. capite, nucha, regione auriculari et gula circumscripte nigris, notæo flavescente cinereofusco, gastræo brunnescente albo hypochondriis ochraceo lavatis, remigibus 1. 2. 3. 8. 9. Sine albedine, 4. 5. 6. 7. Basi utriusque pogonii, 10. 11. 12. 13. 14. Basi pogonii interni albis. Longit. 4 ½ “, alae 2” 1”’, caudae 1” 7”’, rostri a rictu 4”’, tars. 6½”’.

Loxia melanops Natterer Catal. Msc.

Hab. Porto do Rio Araguay.

Ad *Sp. ornatam* (Licht) proxima accedit.

Natterer’s notes: male (in heavy moult, collected at a lake three miles north of Porto do Rio Araguaia, in company of other seedeaters, October). Iris dark brown, yellowish-brown upper bill and tip of lower mandible, the rest of the bill brownish yellow. Length 5¼”, width 7”, the tail reaches 12½”’ over the wingtips.

Whole head, throat, ear-coverts and nape neatly isolated, brilliantly black, remaining upperparts brightly yellowish grey-brown, underside brownish-white becoming ochraceous on the sides, wing and tail feathers deep brown, flight feathers 1. 2. 3. without white, 4. 5. 6. 7. with white base on both vanes, 8. 9. completely without white, 10. 11. 12. 13. 14. with white base in the inner web. The upper wing-covert feathers brownish black with brightly grey-brown edges, the wing and tail feathers edged likewise, and the feathers of the lower half of the eye-ring white.”

[NOTE: *S. ornata* is treated in Hellmayr (1938) as *S. caerulescens ornata*, a subspecies that differs from nominate *caerulescens* in having a dark hood extending to the nape and sides of head. We do not endorse or reject the validity of this subspecies, but point out the taxonomic treatment indicating where the affinities of *S. melanops* were proposed to lie by Pelzeln. Measurements were reported in lines, where 1 line = 2.195 mm]

**B) Sclater’s (1871) description of the type specimen**

Sclater (1871: 21) “*Spermophila melanops*, Pelz. Orn. Bras. pp. 225 et 331.

"Capite, nucha, regione auriculari et gula circumscripte nigris, notæo flavescente cinereo-fusco : gastræo brunnescente albo, hypochondriis ochraceo lavatis: remigibus albo notatis: long. tota 45." *Hab*. Porto do Rio Araguay (*Natt*.). *Obs*. Ad *Sp. caerulescentem* proxime accedit.”

**C) Hellmayr’s (1904) critical description of the type specimen**

Hellmayr (1904: 528) “Oberkopf, Nacken, Kopfseiten und Kehle schwarz, scharf abgesetzt; Rücken gelbligch sandbraun, Flügeldecken, Schwingen und Steuerfedern dunkelbraun mit helleren gelbbräunlichen Aussenrändern. Unterseite lebhaft rahmgelb. Deutliche weisser Flügelspiegel auf der 4.—7. Handschwinge; da auch die ersten Armschwingen weisse Basis besitzen, entsteht ein zweiter Spiegel, der aber von den grossen Flügeldecken und Tertiären verdeckt wird. Schnabel horngelbbraun.

Specimen typicum Mus. Vindob., Nr. 20.316, „♂“ ad., „Rio Araguay, 19. Oktober 1823“, Natterer leg.: a.55, c. 42, r. 9 *mm*.

Diese sehr gut unterschiedene Art steht *S. gutturalis* vielleicht am nächsten, hat wie diese den Kopf ringsum schwarz, allein diese Färbung ist auf dem Nacken sowohl als auf der Kehle scharf abgesetzt, also nicht auf den Vorderhals ausgedehnt, und weicht ferner durch gelblich sandbraunen (statt olivgrünlichen) Rücken und Schwingensäume, rahmgelben (statt blass olivgelben) Unterkörper und hornbraunen (statt weissgelben) Schnabel ab.”

**Translation:** “Cap, nape, side of the head and throat black, sharply confined; back yellowish sandy brown, wing-coverts, remiges and rectrices dark brown with paler yellowish-brown edges. Ventral side brightly cream colored. Clearly white patch on primaries 4–7; as also the secondaries have white bases, a second patch exists, but is hidden by greater coverts and tertials. Bill is horn yellowish brown.

Specimen typicum Mus. Vindob., Nr. 20.316, „♂“ ad., „Rio Araguay, 19. October 1823“, Natterer leg.: a.55, c. 42, r. 9 *mm*.

This well-distinguished species is probably closest to *S. gutturalis*. It is like this one black around its head. On its nape and throat the black is rather restricted; it does not extend to the breast and is different in the yellowish sandy brown back (instead olive-green) and edges of the wings, cream-coloured ventral side (instead of pale olive-yellow) and a horn-brown bill (instead of yellowish white).”

[NOTE: *S. gutturalis* was considered a synonym of *S. nigricollis* by Hellmayr (1938)]

**D) Hellmayr’s (1938) critical description of the type specimen**

Hellmayr (1938: 204) “Sporophila melanops (Pelzeln), a very distinct species, bears some superficial resemblance to *S. n. nigricollis*, but differs at a glance by the following characters. The whole head is black, this color being abruptly defined on the hind neck (instead of passing gradually into the greenish tone of the back) and restricted below to the throat proper (not extending onto the foreneck); back and edges to wings and tail are light brown, between Isabella color and light brownish olive; the under parts from the foreneck down to the tail coverts are dingy buff, between cream buff and chamois (instead of primrose yellow); the tail is shorter; the bill stouter, shorter, with more rounded culmen, and uniform pale brown (instead of marguerite yellow, with plumbeous base). Wing (adult male), 55; tail, 41; bill, 8.

The type obtained by Natterer on October 19, 1823, at Porto do Rio Araguaya, in southern Goyaz, is still unique. Whether Azara's “Pico grueso variable” among other seed-eaters also includes this species, as is assumed by Bertoni (Faun. Parag., p. 65, 1914), remains to be proved by the actual taking of specimens in Paraguay.”

[NOTE: measurements were reported in mm]

**E) Meyer de Schauensee’s (1952) description of the presumed female**

Meyer de Schauensee (1952: 183) “Remarks. —This species apparently rather resembles *S. n. nigricollis*, but differs, among other characters, by having the black of the head sharply defined on the hind neck and restricted below to the throat.

A female from Goiaz which I examined in the collection of the American Museum could possibly belong to this species. Compared to females of *nigricollis*, it is very much browner above, and below it is duller, browner, with no yellowish tint. It measures as follows: wing 57, tail 45, culmen 8 mm., which is slightly larger than the measurements given by Hellmayr. The bill is somewhat more bulky than in *nigricollis*, and the maxilla somewhat more curved.”
