# Supplementary material for: Tiny Bird, Huge Mystery—The Possibly Extinct Hooded Seedeater (Sporophila melanops) Is a Capuchino with a Melanistic Cap
Source: PLoS One. 2016 May 11;11(5):e0154231. doi: 10.1371/journal.pone.0154231 (PMC4864415; doi:10.1371/journal.pone.0154231)
Supplement: S2 Appendix — Most relevant specimens examined for this study. See also other examined specimens in Table 1 and in our previous publications [11,30–32,38,47,49]. (DOCX) [file pone.0154231.s002.docx]

**PLOS One**

**Tiny bird, huge mystery—the Possibly Extinct Hooded Seedeater (*Sporophila melanops*) is a capuchino with a melanistic cap**

Juan Ignacio Areta, Vítor de Q. Piacentini, Elisabeth Haring, Anita Gamauf, Luís Fábio Silveira, Erika Machado, Guy M. Kirwan

**S2 Appendix. Museum specimens examined**. Most relevant specimens examined for this study. See also other examined specimens in Table 1, and our previous publications [11,30–32,38,47,49].

*Sporophila nigricollis*. AMNH 32542, 32543, 32544, 148848, 148849, 16357, 244862, 244865, 244869, 317037 and 514890 (previously erroneously suggested to be a female of *Sporophila melanops*).

*Sporophila castaneiventris*. FMNH 108680, 123990, 154039, 182832, 183952, 183953, 187744, 187745, 190663, 249541, 252360, 252361, 252362, 252363, 252364, 258369, 258370, 258371, 258372, 275713, 275714, 275715, 275716, 275717, 275718, 281453, 281454, 281455, 281456, 282472, 282473, 282474, 282475, 282476, 282477, 282478, 282479, 282480, 282481, 282482, 282483, 282484, 282485, 282486, 282487, 282488, 282489, 282490, 282491, 282492, 282493, 282494, 285003, 287647, 287648, 287649, 287650, 287651, 287652, 287653, 287654, 287655, 287656, 287657, 287658, 287659, 287660, 287661, 287662, 287663, 287664, 287665, 287666, 287667, 287668, 287669, 287670, 287671, 287672, 287673, 287674, 287675, 287676, 287677, 316202, 316509, 316510, 316511, 316512, 316513, 316514, 316515, 316516, 316517, 316518, 316519, 316520, 316521, 316522, 316523, 316524, 316526, 316527, 32405, 344229, 344230, 344231, 346525, 433814, 433815, 433816, 433817, 44396, 44397, 44398, 44399, 4440, 44403, 44404, 49436, 49437, 49438, 49439. LSUMZ 105230, 105264, 105265, 106525, 110882, 110883, 110884, 111607, 111608, 111609, 114524, 116298, 116299, 116300, 120303, 120304, 120305, 120306, 120307, 120308, 120309, 120310, 120664, 120665, 120666, 120667, 120668, 120669, 120670, 120671, 120672, 120673, 120674, 120675, 120676, 120677, 120678, 120679, 120680, 120681, 120682, 120683, 120684, 120685, 120686, 121417, 121418, 121419, 121420, 121421, 121422, 121423, 121424, 121425, 121426, 27758, 28727, 28728, 28729, 34517, 34518, 34519, 34520, 38403, 38404, 38405, 42849, 49028, 50935, 50936, 50937, 50938, 50939, 52493, 52494, 52495, 52496, 62625, 62626, 72908, 83540, 91230, 91231. MPEG 12859, 15142, 18702, 23564, 29013, 31341, 32807, 32808, 32809, 32810, 3527, 3528, 35655, 35656, 37027, 47985, 50211, 50212, 5045, 5046, 56790, 56791, 6080, 6081.

*Sporophila minuta.* FMNH 374143, 374144, 72999, 108676, 108677, 108678, 108679, 110477, 11388, 123981, 123982, 123983, 123984, 123985, 123986, 123987, 123988, 123989, 188883, 191159, 226988, 23561, 249354, 251012, 251472, 251473, 251474, 262026, 262767, 285067, 285068, 289171, 291311, 291312, 291313, 291314, 297831, 297832, 297833, 297834, 298146, 298147, 298148, 298149, 317335, 317336, 32406, 32407, 344227, 344228, 34856, 34857, 34858, 34859, 34860, 34861, 34862, 34863, 34864, 34865, 34866, 34867, 34868, 374145, 374146, 374147, 374148, 374149, 374150, 389267, 389270, 391603, 43797, 43798, 43799, 43800, 49432, 49433, 49434, 49435, 53812, 53813, 72997, 72998. LSUMZ 160798, 160799, 167665, 174838, 175533, 24998, 24999, 27935, 30404, 30405, 30406, 30407, 30408, 39001, 39002, 42350, 48480, 49506, 49515, 49517, 67015, 67016, 67017. MPEG 10988, 15141, 16403, 17407, 17408, 18355, 21650, 21669, 21673, 22517, 22532, 23562, 23563, 25115, 25898, 29011, 29012, 29014, 29015, 30637, 30638, 30639, 30640, 30737, 30738, 30739, 30740, 30741, 32527, 33235, 34396, 34397, 34398, 34399, 39250, 39251, 39252, 39324, 43369, 47221, 50515, 50516, 50517, 50518, 53423, 109541, 109542, 109543, 15778, 15779, 15780, 212670, 289066, 94031, 94032.

*Sporophila ruficollis*. FMNH 152763, 334579, 334580, 334581, 334582, 334583, 334584, 58886, 58887. LSUMZ. 123035, 123036, 125088, 125089, 125090, 125091, 126052, 126053, 126054, 126055, 151415, 151416, 151417, 151418, 151419, 151420, 59756, 59757. MCZ 262172, 86797, 99462.

*Sporophila hypoxantha*. FMNH 152758, 152759, 152761, 152762, 152764, 152765, 152766, 152768, 152770, 152771, 288665, 288666, 296495, 296496, 296497, 296498, 296499, 296500, 296501, 334574, 334575, 334576, 334577, 334578, 335361. LSUMZ 59758, 151408, 169098, 169099, 169100, 169257, 38406, 38407, 59759. MPEG. 47221.

*Sporophila hypochroma.* FMNH 223913. LSUMZ 151407.

*Sporophila cinnamomea.* LSUMZ 34832.
