# Supplementary material for: Tiny Bird, Huge Mystery—The Possibly Extinct Hooded Seedeater (Sporophila melanops) Is a Capuchino with a Melanistic Cap
Source: PLoS One. 2016 May 11;11(5):e0154231. doi: 10.1371/journal.pone.0154231 (PMC4864415; doi:10.1371/journal.pone.0154231)
Supplement: S3 Appendix — Detailed itinerary of the field trips searching for Sporophila melanops in the Araguaia River basin. (DOCX) [file pone.0154231.s003.docx]

**PLOS One**

**Tiny bird, huge mystery—the Possibly Extinct Hooded Seedeater (*Sporophila melanops*) is a capuchino with a melanistic cap**

Juan Ignacio Areta, Vítor de Q. Piacentini, Elisabeth Haring, Anita Gamauf, Luís Fábio Silveira, Erika Machado, Guy M. Kirwan

**S3 Appendix. Field searches.** Detailed itinerary of the field trips searching for *Sporophila melanops* in the Araguaia River basin. Fieldwork was conducted as follows (state abbreviations: GO = Goiás, MT = Mato Grosso, PA = Pará, and TO = Tocantins):

Trip 1, 2008/09: *20–21 December*: drive from Brasília to Chapadão do Céu, GO. *22–23 December*: Emas National Park / Fazenda Santa Amélia. *24 December*: drive to Alto Araguaia, MT / Rio Babilônia, GO. *25 December*: explored road to Araguainha, MT / Rio Babilônia, GO. *26 December*: drive to Barra do Garças, MT / Reserva Ecológica da Serra Azul, MT. *27 December*: Registro do Araguaia, GO (type locality). *28 December*: road to Torixoréu, MT / between Bom Jardim do Goiás and Bazila, GO. *29 December*: Reserva Ecológica da Serra Azul, MT / drive to Aruanã, GO. *30 December*: Aruanã, GO / drive to Gurupi, TO. *31 December*: drive to Caseara, TO. *1–6 January*: Caseara and Araguacema, TO, and Barreira do Campo, PA. *7 January*: Guaraí, TO. *8 January*: between Miranorte and Miracema do Tocantins, TO / drive to Palmas, TO. *9 January*: Aparecida do Rio Negro, TO. *10–11 January*: return to Brasília, checking any suitable areas for *Sporophila* en route.

Trip 2, 2009: *23–24 October*: São Paulo to Barra do Garças, MT. *24–25 October*: Fazenda Eldorado, Barra do Garças, MT. *26 October*: Fazenda Princesa do Araguaia, Registro do Araguaia, GO / drive to Nova Xavantina, MT. *27 October*: “Pantanal do Rio das Mortes”, on road to Cocalinho, MT/ Fazenda Mirante do Araguaia, Cocalinho, MT. *28 October*: road to Peixe, GO / marshland 18 km east of Araguaia River / drive to Lagoa da Confusão, TO. *29–31 October*: around Lagoa da Confusão (including Fazendas Mani, Isadora and Dois Rios). *31 October*: drive to Araguacema, TO. *1–2* *November*: around Araguacema / near Rio Piranhas / and road to Senhor do Bonfim. *3 November*: drive to Imperatriz, Maranhão.

Trip 3, 2010: *2 July*: Brasília to Barra do Garças, MT. *3 July*: road to Água Santa and Registro do Araguaia, GO. *4 July*: road to Serra do Roncador as far as Vale do Sonho, MT. *5 July*: Barra do Garças, MT, to Aruanã, GO, via Araguaiana, Itacaiu and Britânia. *6 July*: Environs of Aruanã, GO. *7 July*: road to Gangas, GO. *8 July*: Aruanã to São Miguel do Araguaia, GO, and road to Luiz Alves, GO. *9 July*: Luiz Alves to Chapéu de Plano, GO. *10 July*: São Miguel do Araguaia, GO, to Lagoa da Confusão, TO, and road to Barreira da Cruz (Rancho Isadora). *11 July*: Rio Urubú and Rancho Isadora, southwest of Lagoa da Confusão, TO. *12 July*: Rancho Isadora and road to Barreira da Cruz, southwest of Lagoa da Confusão, TO. *13 July*: Rancho Isadora, southwest of Lagoa da Confusão, TO, and drive to Anápolis, GO.
