# Supplementary material for: Tiny Bird, Huge Mystery—The Possibly Extinct Hooded Seedeater (Sporophila melanops) Is a Capuchino with a Melanistic Cap
Source: PLoS One. 2016 May 11;11(5):e0154231. doi: 10.1371/journal.pone.0154231 (PMC4864415; doi:10.1371/journal.pone.0154231)
Supplement: S1 Table — Pairwise divergence of the sequences of the male holotype of Sporophila melanops and the presumed female to available sequences of Sporophila in GenBank (as of 29 February 2016). Roman numerals refer to the well-supported clades identified by Mason and Burns [17]. (DOCX) [file pone.0154231.s004.docx]

**PLOS One**

**Tiny bird, huge mystery—the Possibly Extinct Hooded Seedeater (*Sporophila melanops*) is a capuchino with a melanistic cap**

Juan Ignacio Areta, Vítor de Q. Piacentini, Elisabeth Haring, Anita Gamauf, Luís Fábio Silveira, Erika Machado, Guy M. Kirwan

**S1 Table. Genetic *p*-distances for *COI*.** Pairwise divergence of the sequences of the male holotype of *Sporophila melanops* and the presumed female to available sequences of *Sporophila* in GenBank (as of 29 February 2016). Roman numerals refer to the well-supported clades identified by Mason and Burns [17].

| Clade | Species | GenBank number* | Divergence to | |
| --- | --- | --- | --- | --- |
|  |  |  | Holotype | Female |
| I | *Sporophila lineola* | **Spolin1** | 0.0662 | 0.0882 |
| I | *Sporophila lineola* | JN801992 | 0.0662 | 0.0882 |
| II | *Sporophila leucoptera* | KM896574 | 0.0394 | 0.0787 |
| II | *Sporophila leucoptera* | FJ028316 | 0.0397 | 0.0714 |
| III | *Sporophila hypoxantha* | KF316391 | 0.0000 | 0.0690 |
| III | *Sporophila hypoxantha* | KF316390 | 0.0000 | 0.0693 |
| III | *Sporophila hypoxantha* | KF316384 | 0.0000 | 0.0636 |
| III | *Sporophila hypoxantha* | GU070603 | 0.0000 | 0.0787 |
| III | *Sporophila hypoxantha* | GU070601 | 0.0000 | 0.0614 |
| III | *Sporophila hypoxantha* | GU070600 | 0.0000 | 0.0707 |
| III | *Sporophila hypoxantha* | FJ028312 | 0.0000 | 0.0720 |
| III | *Sporophila melanogaster* | GU070609 | 0.0000 | 0.0761 |
| III | *Sporophila melanogaster* | GU070608 | 0.0000 | 0.0795 |
| III | *Sporophila melanogaster* | GU070607 | 0.0000 | 0.0795 |
| III | *Sporophila palustris* | KF316399 | 0.0000 | 0.0700 |
| III | *Sporophila pileata* | GU070599 | 0.0000 | 0.0631 |
| III | *Sporophila pileata* | GU070598 | 0.0000 | 0.0680 |
| III | *Sporophila pileata* | GU070597 | 0.0000 | 0.0693 |
| III | *Sporophila pileata* | GU070594 | 0.0000 | 0.0693 |
| III | *Sporophila ruficollis* | GU070613 | 0.0000 | 0.0693 |
| III | *Sporophila ruficollis* | FJ028320 | 0.0000 | 0.0794 |
| III | *Sporophila ruficollis* | FJ028319 | 0.0000 | 0.0636 |
| III | *Sporophila bouvreuil* | JN801989 | 0.0074 | 0.0809 |
| III | *Sporophila melanogaster* | **Spomel2** | 0.0074 | 0.0809 |
| III | *Sporophila melanogaster* | **Spomel1** | 0.0074 | 0.0809 |
| III | *Sporophila palustris* | **Spopal1** | 0.0074 | 0.0809 |
| III | *Sporophila hypoxantha* | GU070604 | 0.0078 | 0.0775 |
| III | *Sporophila melanogaster* | GU070610 | 0.0078 | 0.0775 |
| III | *Sporophila melanogaster* | GU070605 | 0.0078 | 0.0775 |
| III | *Sporophila bouvreuil* | KF316375 | 0.0079 | 0.0787 |
| III | *Sporophila bouvreuil* | KF316374 | 0.0079 | 0.0787 |
| III | *Sporophila bouvreuil* | KF316373 | 0.0079 | 0.0787 |
| III | *Sporophila bouvreuil* | KF316372 | 0.0079 | 0.0787 |
| III | *Sporophila bouvreuil* | KF316371 | 0.0079 | 0.0787 |
| III | *Sporophila bouvreuil* | KF316370 | 0.0079 | 0.0787 |
| III | *Sporophila bouvreuil* | KF316368 | 0.0079 | 0.0787 |
| III | *Sporophila bouvreuil* | KF316367 | 0.0079 | 0.0787 |
| III | *Sporophila bouvreuil* | KF316366 | 0.0079 | 0.0787 |
| III | *Sporophila bouvreuil* | KF316365 | 0.0079 | 0.0787 |
| III | *Sporophila bouvreuil* | KF316364 | 0.0079 | 0.0787 |
| III | *Sporophila bouvreuil* | KF316363 | 0.0079 | 0.0787 |
| III | *Sporophila bouvreuil* | KF316362 | 0.0079 | 0.0787 |
| III | *Sporophila bouvreuil* | KF316361 | 0.0079 | 0.0787 |
| III | *Sporophila bouvreuil* | KF316360 | 0.0079 | 0.0787 |
| III | *Sporophila bouvreuil* | KF316359 | 0.0079 | 0.0787 |
| III | *Sporophila cinnamomea* | KF316376 | 0.0079 | 0.0787 |
| III | *Sporophila cinnamomea* | JQ176250 | 0.0079 | 0.0787 |
| III | *Sporophila cinnamomea* | JQ176249 | 0.0079 | 0.0787 |
| III | *Sporophila cinnamomea* | FJ028305 | 0.0079 | 0.0787 |
| III | *Sporophila hypochroma* | KF316377 | 0.0079 | 0.0787 |
| III | *Sporophila hypochroma* | FJ028311 | 0.0079 | 0.0787 |
| III | *Sporophila hypoxantha* | KF316398 | 0.0079 | 0.0787 |
| III | *Sporophila hypoxantha* | KF316394 | 0.0079 | 0.0787 |
| III | *Sporophila hypoxantha* | KF316393 | 0.0079 | 0.0787 |
| III | *Sporophila hypoxantha* | KF316389 | 0.0079 | 0.0787 |
| III | *Sporophila hypoxantha* | KF316387 | 0.0079 | 0.0787 |
| III | *Sporophila hypoxantha* | KF316386 | 0.0079 | 0.0787 |
| III | *Sporophila hypoxantha* | KF316385 | 0.0079 | 0.0787 |
| III | *Sporophila hypoxantha* | KF316382 | 0.0079 | 0.0787 |
| III | *Sporophila hypoxantha* | KF316381 | 0.0079 | 0.0787 |
| III | *Sporophila hypoxantha* | FJ028314 | 0.0079 | 0.0787 |
| III | *Sporophila hypoxantha* | FJ028313 | 0.0079 | 0.0787 |
| III | *Sporophila palustris* | KF316403 | 0.0079 | 0.0787 |
| III | *Sporophila palustris* | KF316402 | 0.0079 | 0.0787 |
| III | *Sporophila palustris* | KF316401 | 0.0079 | 0.0787 |
| III | *Sporophila palustris* | KF316400 | 0.0079 | 0.0787 |
| III | *Sporophila palustris* | FJ028318 | 0.0079 | 0.0787 |
| III | *Sporophila palustris* | FJ028317 | 0.0079 | 0.0787 |
| III | *Sporophila palustris "zelichi"* | FJ028322 | 0.0079 | 0.0787 |
| III | *Sporophila pileata* | KF316357 | 0.0079 | 0.0787 |
| III | *Sporophila pileata* | KF316356 | 0.0079 | 0.0787 |
| III | *Sporophila pileata* | KF316355 | 0.0079 | 0.0787 |
| III | *Sporophila pileata* | KF316354 | 0.0079 | 0.0787 |
| III | *Sporophila ruficollis* | FJ028321 | 0.0079 | 0.0787 |
| III | *Sporophila hypoxantha* | KF316392 | 0.0079 | 0.0794 |
| III | *Sporophila cinnamomea* | FJ028304 | 0.0080 | 0.0800 |
| III | *Sporophila hypoxantha* | KF316396 | 0.0080 | 0.0800 |
| III | *Sporophila melanogaster* | GU070611 | 0.0081 | 0.0726 |
| III | *Sporophila hypoxantha* | KF316378 | 0.0091 | 0.0727 |
| III | *Sporophila hypoxantha* | KF316380 | 0.0099 | 0.0792 |
| III | *Sporophila melanogaster* | GU070606 | 0.0099 | 0.0792 |
| III | *Sporophila pileata* | GU070595 | 0.0100 | 0.0800 |
| III | *Sporophila hypoxantha* | Spohyp-1 | 0.0147 | 0.0882 |
| III | *Sporophila bouvreuil* | KF316369 | 0.0157 | 0.0866 |
| III | *Sporophila bouvreuil* | KF316358 | 0.0157 | 0.0866 |
| III | *Sporophila hypoxantha* | KF316395 | 0.0157 | 0.0866 |
| III | *Sporophila hypoxantha* | KF316379 | 0.0157 | 0.0866 |
| III | *Sporophila hypoxantha* | FJ028315 | 0.0157 | 0.0866 |
| III | *Sporophila hypoxantha* | KF316397 | 0.0159 | 0.0635 |
| III | *Sporophila minuta* | GU070591 | 0.0194 | 0.0680 |
| III | *Sporophila minuta* | GU070590 | 0.0196 | 0.0686 |
| III | *Sporophila minuta* | GU070587 | 0.0198 | 0.0693 |
| III | *Sporophila minuta* | GU070589 | 0.0220 | 0.0769 |
| III | *Sporophila minuta* | GU070586 | 0.0227 | 0.0795 |
| III | *Sporophila minuta* | JQ176257 | 0.0236 | 0.0787 |
| III | *Sporophila minuta* | JQ176255 | 0.0236 | 0.0787 |
| III | *Sporophila minuta* | GU070592 | 0.0236 | 0.0787 |
| III | *Sporophila minuta* | **Spominmin2** | 0.0294 | 0.0735 |
| III | *Sporophila minuta* | **Spominmin1** | 0.0294 | 0.0735 |
| III | *Sporophila minuta* | JQ176256 | 0.0309 | 0.0825 |
| III | *Sporophila minuta* | KM894380 | 0.0417 | 0.0833 |
| III | *Sporophila castaneiventris* | GU070584 | 0.0481 | 0.1154 |
| III | *Sporophila castaneiventris* | GU070585 | 0.0485 | 0.1165 |
| III | *Sporophila minuta* | DQ434138 | 0.0543 | 0.0775 |
| III | *Sporophila minuta* | DQ434137 | 0.0543 | 0.0775 |
| III | *Sporophila* "*torqueola*"  [= *minuta*] | KM894383 | 0.0556 | 0.0926 |
| III | *Sporophila minuta* | KM894378 | 0.0565 | 0.0806 |
| III | *Sporophila minuta* | KM894377 | 0.0565 | 0.0806 |
| III | *Sporophila castaneiventris* | GU070583 | 0.0620 | 0.1163 |
| IV | *Oryzoborus crassirostris* | JQ175642 | 0.0420 | 0.0588 |
| IV | *Oryzoborus angolensis* | JQ175641 | 0.0630 | 0.0866 |
| IV | *Oryzoborus angolensis* | FJ027930 | 0.0630 | 0.0866 |
| IV | *Oryzoborus angolensis* | FJ027929 | 0.0630 | 0.0866 |
| IV | *Oryzoborus angolensis* | FJ027928 | 0.0630 | 0.0866 |
| IV | *Oryzoborus crassirostris* | JQ175644 | 0.0630 | 0.0551 |
| IV | *Oryzoborus crassirostris* | JQ175643 | 0.0630 | 0.0551 |
| IV | *Oryzoborus angolensis* | KM896483 | 0.0709 | 0.0945 |
| V | *Sporophila intermedia* | JQ176254 | 0.0787 | 0.0551 |
| V | *Sporophila intermedia* | JQ176253 | 0.0787 | 0.0551 |
| V | *Sporophila intermedia* | JQ176252 | 0.0787 | 0.0551 |
| V | *Sporophila americana* | JQ176248 | 0.0866 | 0.0630 |
| V | *Sporophila americana* | JQ176247 | 0.0866 | 0.0630 |
| V | *Sporophila americana* | JQ176246 | 0.0866 | 0.0630 |
| VI | *Sporophila torqueola* | KM894381 | 0.0645 | 0.0887 |
| VI | *Sporophila* "*minuta*"  [= *torqueola*] | KM894379 | 0.0787 | 0.0945 |
| VI | *Sporophila torqueola* | KM894384 | 0.0787 | 0.0945 |
| VI | *Sporophila torqueola* | KM894382 | 0.0787 | 0.0945 |
| VI | *Sporophila torqueola* | JN801378 | 0.0787 | 0.0945 |
| VII | *Dolospingus fringilloides* | JQ174710 | 0.0614 | 0.0351 |
| VII | *Sporophila nigricollis* | JQ176258 | 0.0787 | 0.0236 |
| VII | *Sporophila nigricollis* | KM896576 | 0.0794 | 0.0238 |
| VII | *Sporophila nigricollis* | KM896575 | 0.0794 | 0.0238 |
| VII | *Sporophila nigricollis* | JN801994 | 0.0809 | 0.0221 |
| VII | *Sporophila nigricollis* | JN801993 | 0.0809 | 0.0221 |
| VII | *Sporophila nigricollis* | **Sponignig4** | 0.0809 | 0.0221 |
| VII | *Sporophila nigricollis* | **Sponignig3** | 0.0809 | 0.0221 |
| VII | *Sporophila nigricollis* | **Sponignig1** | 0.0809 | 0.0221 |
| VII | *Sporophila caerulescens* | JQ627352 | 0.0855 | 0.0171 |
| VII | *Dolospingus fringilloides* | JQ174709 | 0.0866 | 0.0472 |
| VII | *Sporophila caerulescens* | KM896572 | 0.0866 | 0.0000 |
| VII | *Sporophila caerulescens* | FJ028303 | 0.0866 | 0.0000 |
| VII | *Sporophila caerulescens* | FJ028302 | 0.0866 | 0.0000 |
| VII | *Sporophila caerulescens* | FJ028301 | 0.0866 | 0.0000 |
| VII | *Sporophila caerulescens* | FJ028300 | 0.0866 | 0.0000 |
| VII | *Sporophila caerulescens* | FJ028299 | 0.0866 | 0.0000 |
| VII | *Sporophila caerulescens* | FJ028298 | 0.0866 | 0.0000 |
| VII | *Sporophila caerulescens* | **Spocaecae1** | 0.0882 | 0.0000 |
| VII | *Sporophila caerulescens* | JN801990 | 0.0882 | 0.0000 |
| VII | *Sporophila luctuosa* | **Spoluc1** | 0.0882 | 0.0147 |
| VII | *Sporophila nigricollis* | **Sponignig2** | 0.0882 | 0.0000 |
| VII | *Sporophila nigricollis* | JQ176259 | 0.1024 | 0.0157 |
| VII | *Sporophila nigricollis vivida* | **Sponigviv1** | 0.1029 | 0.0294 |
| VIII | *Sporophila schistacea* | JQ176264 | 0.0417 | 0.0583 |
| IX | *Sporophila collaris* | FJ028306 | 0.0412 | 0.0619 |
| IX | *Sporophila collaris* | FJ028307 | 0.0476 | 0.0556 |
| IX | *Sporophila collaris* | JQ176251 | 0.0551 | 0.0551 |
| IX | *Sporophila collaris* | FJ028310 | 0.0551 | 0.0551 |
| IX | *Sporophila collaris* | FJ028309 | 0.0551 | 0.0551 |
| IX | *Sporophila collaris* | FJ028308 | 0.0551 | 0.0551 |
| IX | *Sporophila collaris* | JN801991 | 0.0588 | 0.0515 |
| IX | *Sporophila plumbea* | JQ176262 | 0.0630 | 0.0630 |
| IX | *Sporophila plumbea* | JQ176261 | 0.0630 | 0.0551 |
| IX | *Sporophila plumbea* | JQ176260 | 0.0630 | 0.0551 |
| IX | *Sporophila collaris* | KM896573 | 0.0709 | 0.0630 |
| IX | *Sporophila albogularis* | **Spoalb-1** | 0.0882 | 0.0735 |

* Sequences in bold refer to our own data (see Table 1 in text and S3 Table).
