# Supplementary material for: Tiny Bird, Huge Mystery—The Possibly Extinct Hooded Seedeater (Sporophila melanops) Is a Capuchino with a Melanistic Cap
Source: PLoS One. 2016 May 11;11(5):e0154231. doi: 10.1371/journal.pone.0154231 (PMC4864415; doi:10.1371/journal.pone.0154231)
Supplement: S2 Table — Pairwise divergence of the sequences of the male holotype of Sporophila melanops and the presumed female to available sequences of Sporophila in GenBank (as of 29 February 2016). Roman numerals refer to the well-supported clades identified by Mason and Burns [17]. (DOCX) [file pone.0154231.s005.docx]

**PLOS One**

**Tiny bird, huge mystery—the Possibly Extinct Hooded Seedeater (*Sporophila melanops*) is a capuchino with a melanistic cap**

Juan Ignacio Areta, Vítor de Q. Piacentini, Elisabeth Haring, Anita Gamauf, Luís Fábio Silveira, Erika Machado, Guy M. Kirwan

**S2 Table. Genetic *p*-distances for *Cyt-b*.** Pairwise divergence of the sequences of the male holotype of *Sporophila melanops* and the presumed female to available sequences of *Sporophila* in GenBank (as of 29 February 2016). Roman numerals refer to the well-supported clades identified by Mason and Burns [17].

| Clade | Species | GenBank number* | Divergence to | |
| --- | --- | --- | --- | --- |
|  |  |  | Holotype | Female |
| I | *Sporophila lineola* | JN810142 | 0.1308 | 0.1154 |
| I | *Sporophila lineola* | **Spolin1** | 0.1308 | 0.1154 |
| II | *Sporophila leucoptera* | JN677025 | 0.0306 | 0.0306 |
| II | *Sporophila simplex* | JN810149 | 0.0462 | 0.0308 |
| II | *Sporophila telasco* | JN810150 | 0.0462 | 0.0308 |
| II | *Sporophila leucoptera* | JN810141 | 0.0538 | 0.0615 |
| II | *Sporophila leucoptera* | AY387431 | 0.0556 | 0.0397 |
| II | *Sporophila peruviana* | JN810147 | 0.0615 | 0.0462 |
| III | *Sporophila bouvreuil* | KF234241 | 0.0000 | 0.0308 |
| III | *Sporophila bouvreuil* | JN676994 | 0.0000 | 0.0256 |
| III | *Sporophila cinnamomea* | KF316448 | 0.0000 | 0.0198 |
| III | *Sporophila cinnamomea* | JN810134 | 0.0000 | 0.0308 |
| III | *Sporophila cinnamomea* | JN677008 | 0.0000 | 0.0196 |
| III | *Sporophila cinnamomea* | JN677007 | 0.0000 | 0.0196 |
| III | *Sporophila cinnamomea* | AY387423 | 0.0000 | 0.0317 |
| III | *Sporophila cinnamomea* | AY387422 | 0.0000 | 0.0317 |
| III | *Sporophila cinnamomea* | AY387421 | 0.0000 | 0.0317 |
| III | *Sporophila cinnamomea* | AY387420 | 0.0000 | 0.0317 |
| III | *Sporophila hypochroma* | KF316449 | 0.0000 | 0.0198 |
| III | *Sporophila hypochroma* | JN810139 | 0.0000 | 0.0308 |
| III | *Sporophila hypochroma* | AY387428 | 0.0000 | 0.0317 |
| III | *Sporophila hypochroma* | AY387427 | 0.0000 | 0.0317 |
| III | *Sporophila hypoxantha* | KF316470 | 0.0000 | 0.0196 |
| III | *Sporophila hypoxantha* | KF316469 | 0.0000 | 0.0233 |
| III | *Sporophila hypoxantha* | KF316468 | 0.0000 | 0.0196 |
| III | *Sporophila hypoxantha* | KF316466 | 0.0000 | 0.0233 |
| III | *Sporophila hypoxantha* | KF316464 | 0.0000 | 0.0263 |
| III | *Sporophila hypoxantha* | KF316463 | 0.0000 | 0.0263 |
| III | *Sporophila hypoxantha* | KF316462 | 0.0000 | 0.0317 |
| III | *Sporophila hypoxantha* | KF316460 | 0.0000 | 0.0202 |
| III | *Sporophila hypoxantha* | KF316459 | 0.0000 | 0.0263 |
| III | *Sporophila hypoxantha* | KF316458 | 0.0000 | 0.0263 |
| III | *Sporophila hypoxantha* | KF316454 | 0.0000 | 0.0263 |
| III | *Sporophila hypoxantha* | KF316453 | 0.0000 | 0.0235 |
| III | *Sporophila hypoxantha* | JN810140 | 0.0000 | 0.0308 |
| III | *Sporophila hypoxantha* | JN677023 | 0.0000 | 0.0222 |
| III | *Sporophila hypoxantha* | JN677021 | 0.0000 | 0.0187 |
| III | *Sporophila hypoxantha* | JN677020 | 0.0000 | 0.0187 |
| III | *Sporophila hypoxantha* | JN677019 | 0.0000 | 0.0256 |
| III | *Sporophila hypoxantha* | JN677018 | 0.0000 | 0.0187 |
| III | *Sporophila hypoxantha* | JN677017 | 0.0000 | 0.0187 |
| III | *Sporophila hypoxantha* | JN677016 | 0.0000 | 0.0187 |
| III | *Sporophila hypoxantha* | JN677015 | 0.0000 | 0.0198 |
| III | *Sporophila hypoxantha* | AY387430 | 0.0000 | 0.0317 |
| III | *Sporophila hypoxantha* | AY387429 | 0.0000 | 0.0317 |
| III | *Sporophila hypoxantha* | **Spohyp1** | 0.0000 | 0.0308 |
| III | *Sporophila melanogaster* | JN810144 | 0.0000 | 0.0308 |
| III | *Sporophila melanogaster* | JN677032 | 0.0000 | 0.0196 |
| III | *Sporophila melanogaster* | JN677031 | 0.0000 | 0.0196 |
| III | *Sporophila melanogaster* | JN677030 | 0.0000 | 0.0196 |
| III | *Sporophila melanogaster* | JN677029 | 0.0000 | 0.0196 |
| III | *Sporophila melanogaster* | JN677028 | 0.0000 | 0.0196 |
| III | *Sporophila melanogaster* | JN677027 | 0.0000 | 0.0196 |
| III | *Sporophila melanogaster* | JN677026 | 0.0000 | 0.0189 |
| III | *Sporophila melanogaster* | **Spomel2** | 0.0000 | 0.0308 |
| III | *Sporophila palustris* | **Spopal1** | 0.0000 | 0.0308 |
| III | *Sporophila palustris* | KF316471 | 0.0000 | 0.0233 |
| III | *Sporophila palustris* | JN810146 | 0.0000 | 0.0308 |
| III | *Sporophila palustris* | JN677040 | 0.0000 | 0.0196 |
| III | *Sporophila palustris* | AY387439 | 0.0000 | 0.0317 |
| III | *Sporophila palustris* | AY387438 | 0.0000 | 0.0317 |
| III | *Sporophila palustris* “zelichi” | JN677050 | 0.0000 | 0.0233 |
| III | *Sporophila palustris* “zelichi” | AY387445 | 0.0000 | 0.0317 |
| III | *Sporophila palustris* “zelichi” | AY387444 | 0.0000 | 0.0317 |
| III | *Sporophila pileata* | JN810131 | 0.0000 | 0.0308 |
| III | *Sporophila pileata* | JN676997 | 0.0000 | 0.0187 |
| III | *Sporophila pileata* | JN676996 | 0.0000 | 0.0187 |
| III | *Sporophila pileata* | JN676992 | 0.0000 | 0.0187 |
| III | *Sporophila pileata* | JN676991 | 0.0000 | 0.0187 |
| III | *Sporophila ruficollis* | JN677047 | 0.0000 | 0.0233 |
| III | *Sporophila ruficollis* | JN677046 | 0.0000 | 0.0194 |
| III | *Sporophila ruficollis* | JN677045 | 0.0000 | 0.0194 |
| III | *Sporophila ruficollis* | JN677044 | 0.0000 | 0.0187 |
| III | *Sporophila ruficollis* | JN677043 | 0.0000 | 0.0187 |
| III | *Sporophila ruficollis* | AY387442 | 0.0000 | 0.0317 |
| III | *Sporophila ruficollis* | AY387441 | 0.0000 | 0.0317 |
| III | *Sporophila ruficollis* | AY387440 | 0.0000 | 0.0317 |
| III | *Sporophila ruficollis* | AF489896 | 0.0000 | 0.0308 |
| III | *Sporophila pileata* | AY387415 | 0.0079 | 0.0397 |
| III | *Sporophila hypoxantha* | KF316461 | 0.0088 | 0.0351 |
| III | *Sporophila hypoxantha* | KF316465 | 0.0092 | 0.0367 |
| III | *Sporophila pileata* | JN676995 | 0.0093 | 0.0280 |
| III | *Sporophila pileata* | JN676993 | 0.0093 | 0.0280 |
| III | *Sporophila palustris* | JN677042 | 0.0097 | 0.0291 |
| III | *Sporophila hypochroma* | JN677014 | 0.0098 | 0.0294 |
| III | *Sporophila ruficollis* | JN677049 | 0.0098 | 0.0294 |
| III | *Sporophila palustris* | JN677041 | 0.0116 | 0.0349 |
| III | *Sporophila palustris* | KF316472 | 0.0118 | 0.0353 |
| III | *Sporophila melanogaster* | **Spomel1** | 0.0154 | 0.0462 |
| III | *Sporophila minuta* | GU215337 | 0.0154 | 0.0462 |
| III | *Sporophila pileata* | AY387414 | 0.0159 | 0.0476 |
| III | *Sporophila castaneiventris* | AY387419 | 0.0159 | 0.0476 |
| III | *Sporophila melanogaster* | AY387433 | 0.0159 | 0.0476 |
| III | *Sporophila minuta* | AY387435 | 0.0159 | 0.0476 |
| III | *Sporophila minuta* | JN677033 | 0.0198 | 0.0396 |
| III | *Sporophila minuta* | JN810145 | 0.0231 | 0.0538 |
| III | *Sporophila minuta* | GU215338 | 0.0231 | 0.0538 |
| III | *Sporophila castaneiventris* | AY387418 | 0.0238 | 0.0556 |
| III | *Sporophila minuta* | AY387436 | 0.0238 | 0.0556 |
| III | *Sporophila minuta* | AY387434 | 0.0238 | 0.0556 |
| III | *Sporophila castaneiventris* | JN677006 | 0.0297 | 0.0297 |
| III | *Sporophila castaneiventris* | JN677005 | 0.0297 | 0.0297 |
| III | *Sporophila minuta* | JN677039 | 0.0297 | 0.0495 |
| III | *Sporophila minuta* | JN677038 | 0.0297 | 0.0495 |
| III | *Sporophila minuta* | JN677037 | 0.0297 | 0.0495 |
| III | *Sporophila minuta* | JN677036 | 0.0297 | 0.0495 |
| III | *Sporophila minuta* | JN677035 | 0.0297 | 0.0495 |
| III | *Sporophila minuta* | JN677034 | 0.0297 | 0.0495 |
| III | *Sporophila castaneiventris* | **Spocas1** | 0.0462 | 0.0538 |
| III | *Sporophila castaneiventris* | JN677004 | 0.0490 | 0.0490 |
| III | *Sporophila castaneiventris* | JN810133 | 0.0538 | 0.0462 |
| III | *Sporophila castaneiventris* | AF310056 | 0.0556 | 0.0476 |
| IV | *Oryzoborus nuttingi* | JN810099 | 0.0308 | 0.0154 |
| V | *Sporophila intermedia* | EU647922 | 0.0462 | 0.0308 |
| V | *Sporophila americana* | AF310054 | 0.0556 | 0.0397 |
| V | *Sporophila corvina* | JN810136 | 0.0385 | 0.0462 |
| V | *Sporophila corvina* | GU215335 | 0.0385 | 0.0462 |
| V | *Sporophila corvina* | GU215336 | 0.0462 | 0.0538 |
| VI | *Sporophila torqueola* | JN810151 | 0.0462 | 0.0308 |
| VII | *Sporophila nigricollis* | AF310053 | 0.0159 | 0.0238 |
| VII | *Sporophila caerulescens* | JN677001 | 0.0196 | 0.0000 |
| VII | *Sporophila caerulescens* | JN677000 | 0.0196 | 0.0000 |
| VII | *Sporophila caerulescens* | JN677002 | 0.0227 | 0.0000 |
| VII | *Dolospingus fringilloides* | JN810073 | 0.0231 | 0.0231 |
| VII | *Dolospingus fringilloides* | AY705434 | 0.0231 | 0.0231 |
| VII | *Dolospingus fringilloides* | AY705435 | 0.0231 | 0.0231 |
| VII | *Sporophila caerulescens* | **Spocaecae1** | 0.0308 | 0.0000 |
| VII | *Sporophila caerulescens* | JN810132 | 0.0308 | 0.0000 |
| VII | *Sporophila n. nigricollis* | **Sponignig1** | 0.0308 | 0.0000 |
| VII | *Sporophila n. nigricollis* | **Sponignig3** | 0.0308 | 0.0000 |
| VII | *Sporophila nigricollis* | GU215339 | 0.0313 | 0.0000 |
| VII | *Sporophila caerulescens* | AY387417 | 0.0317 | 0.0000 |
| VII | *Sporophila caerulescens* | AY387416 | 0.0317 | 0.0000 |
| VII | *Sporophila nigricollis* | AY387437 | 0.0317 | 0.0000 |
| VII | *Sporophila luctuosa* | JN810143 | 0.0462 | 0.0308 |
| VII | *Sporophila luctuosa* | AY387432 | 0.0476 | 0.0317 |
| VIII | *Sporophila schistacea* | AF290149 | 0.0370 | 0.0370 |
| VIII | *Sporophila schistacea* | GU215340 | 0.0620 | 0.0465 |
| VIII | *Sporophila falcirostris* | AY387425 | 0.0714 | 0.0556 |
| VIII | *Sporophila falcirostris* | JN810137 | 0.0769 | 0.0615 |
| VIII | *Sporophila schistacea* | EF529976 | 0.0769 | 0.0615 |
| VIII | *Sporophila falcirostris* | AY387426 | 0.0794 | 0.0635 |
| IX | *Sporophila collaris* | JN677013 | 0.0185 | 0.0185 |
| IX | *Sporophila collaris* | JN677012 | 0.0185 | 0.0185 |
| IX | *Sporophila collaris* | JN677010 | 0.0194 | 0.0194 |
| IX | *Sporophila collaris* | JN677011 | 0.0196 | 0.0196 |
| IX | *Sporophila collaris* | JN677009 | 0.0196 | 0.0196 |
| IX | *Sporophila collaris* | KP965518 | 0.0215 | 0.0215 |
| IX | *Sporophila collaris* | AF489895 | 0.0348 | 0.0261 |
| IX | *Sporophila albogularis* | JN810130 | 0.0538 | 0.0538 |
| IX | *Sporophila albogularis* | **Spoalb1** | 0.0538 | 0.0538 |
| IX | *Sporophila plumbea* | JN810148 | 0.0615 | 0.0462 |
| IX | *Sporophila collaris* | JN810135 | 0.0692 | 0.0538 |
| *S. frontalis* | *Sporophila frontalis* | JN810138 | 0.0446 | 0.0446 |

* Sequences in bold refer to our own data (see Table 1 in text and S3 Table).
