# Supplementary material for: Tiny Bird, Huge Mystery—The Possibly Extinct Hooded Seedeater (Sporophila melanops) Is a Capuchino with a Melanistic Cap
Source: PLoS One. 2016 May 11;11(5):e0154231. doi: 10.1371/journal.pone.0154231 (PMC4864415; doi:10.1371/journal.pone.0154231)
Supplement: S3 Table — Short fragments (<200 bp) of COI and Cyt-b genes of specimens sequenced for the present study. (DOCX) [file pone.0154231.s006.docx]

**PLOS One**

**Tiny bird, huge mystery—the Possibly Extinct Hooded Seedeater (*Sporophila melanops*) is a capuchino with a melanistic cap**

Juan Ignacio Areta, Vítor de Q. Piacentini, Elisabeth Haring, Anita Gamauf, Luís Fábio Silveira, Erika Machado, Guy M. Kirwan

**S3 Table. Genetic sequences.** Short fragments (<200 bp) of *COI* and *Cyt-b* genes of specimens sequenced for the present study.

*Sporophila melanops* (**holotype of *S. melanops***), Spomps1

Cyt-b

CTTCGGATCGCTCCTGGGCGTCTGCTTAATTACTCAAATCGTAACAGGCCTACTGCTGGCCATACACTATACAGCAGACACCAGCCTGGCCTTCTCTTCTGTCGCCCACATATGCCGAGACGTACAATTC

COI

CATCGGAACCCTATATTTAATCTTCGGCGCATGAGCCGGAATAGTAGGTACCGCCCTAAGCCTCCTCATCCGAGCAGAACTAGGCCAACCTGGAGCCCTTCTAGGAGACGACCAAGTCTACAACGTAGTCGTCACA

*Sporophila* sp. (**presumed *S. melanops* female**), Spomps2

Cyt-b

CTTCGGATCGCTACTGGGCGTTTGCTTAATTACTCAAATCGTAACAGGCCTACTGCTGGCCATACACTATACAGCAGACACCAGCTTGGCCTTCTCCTCTGTCGCCCACATATGCCGAGACGTACAATTC

COI

CATTGGAACCCTGTACCTAATTTTCGGCGCATGAGCCGGAATAGTAGGTACCGCCCTAAGCCTCCTTATCCGAGCAGAGCTAGGCCAACCCGGAGCCCTCCTCGGAGACGACCAAGTTTACAACGTAGTCGTTACA

*Sporophila n. nigricollis*, Sponignig1

Cyt-b

CTTCGGATCGCTACTGGGCGTTTGCTTAATTACTCAAATCGTAACAGGCCTACTGCTGGCCATACACTATACAGCAGACACCAGCTTGGCCTTCTCCTCTGTCGCCCACATATGCCGAGACGTACAATTC

COI

CATTGGAACCCTGTACCTAATTTTCGGTGCATGAGCCGGAATAGTAGGTACCGCCCTAAGCCTCCTTATCCGAGCAGAACTAGGCCAACCTGGAGCCCTCCTCGGAGACGACCAAGTTTACAACGTAGTCGTTACA

*Sporophila n. nigricollis*, Sponignig2

COI

CATTGGAACCCTGTACCTAATTTTCGGCGCATGAGCCGGAATAGTAGGTACCGCCCTAAGCCTCCTTATCCGAGCAGAGCTAGGCCAACCCGGAGCCCTCCTCGGAGACGACCAAGTTTACAACGTAGTCGTTACA

*Sporophila n. nigricollis*, Sponignig3

Cyt-b

CTTCGGATCGCTACTGGGCGTTTGCTTAATTACTCAAATCGTAACAGGCCTACTGCTGGCCATACACTATACAGCAGACACCAGCTTGGCCTTCTCCTCTGTCGCCCACATATGCCGAGACGTACAATTC

COI

CATTGGAACCCTGTACCTAATTTTCGGTGCATGAGCCGGAATAGTAGGTACCGCCCTAAGCCTCCTTATCCGAGCAGAACTAGGCCAACCTGGAGCCCTCCTCGGAGACGACCAAGTTTACAACGTAGTCGTTACA

*Sporophila n. nigricollis*, Sponignig4

COI

CATTGGAACCCTGTACCTAATTTTCGGTGCATGAGCCGGAATAGTAGGTACCGCCCTAAGCCTCCTTATCCGAGCAGAACTAGGCCAACCTGGAGCCCTCCTCGGAGACGACCAAGTTTACAACGTAGTCGTTACA

*Sporophila nigricollis vivida*, Sponigviv1

COI

CATTGGAACCCTGTACCTAATTTTCGGTGCATGAGCCGGAATAGTAGGTACCGCCCTAAGCCTCCTTATCCGGGCAGAGCTAGGCCAACCTGGAGCCCTCCTCGGAGACGACCAAGTTTACAACGTAATCGTTACA

*Sporophila caerulescens*, Spocaecae1

Cyt-b

CTTCGGATCGCTACTGGGCGTTTGCTTAATTACTCAAATCGTAACAGGCCTACTGCTGGCCATACACTATACAGCAGACACCAGCTTGGCCTTCTCCTCTGTCGCCCACATATGCCGAGACGTACAATTC

COI

CATTGGAACCCTGTACCTAATTTTCGGCGCATGAGCCGGAATAGTAGGTACCGCCCTAAGCCTCCTTATCCGAGCAGAGCTAGGCCAACCCGGAGCCCTCCTCGGAGACGACCAAGTTTACAACGTAGTCGTTACA

*Sporophila palustris* (holotype of *S. lorenzi*)*,* Spopal1

Cyt-b

CTTCGGATCGCTCCTGGGCGTCTGCTTAATTACTCAAATCGTAACAGGCCTACTGCTGGCCATACACTATACAGCAGACACCAGCCTGGCCTTCTCTTCTGTCGCCCACATATGCCGAGACGTACAATTC

COI

CATCGGAACCCTATACTTAATCTTCGGCGCATGAGCCGGAATAGTAGGTACCGCCCTAAGCCTCCTCATCCGAGCAGAACTAGGCCAACCTGGAGCCCTTCTAGGAGACGACCAAGTCTACAACGTAGTCGTCACA

*Sporophila melanogaster* (**syntype**), Spomel1

Cyt-b

CTTCGGATCGTTCCTGGGCGTCTGCTTAATTACTCAAATCGTAACAGGCCTACTGCTGGCCATACACTATACAGCAGACACCAGCCTGGCTTTCTCTTCTGTCGCCCACATATGCCGAGACGTACAATTC

COI

CATCGGAACCCTATACTTAATCTTCGGCGCATGAGCCGGAATAGTAGGTACCGCCCTAAGCCTCCTCATCCGAGCAGAACTAGGCCAACCTGGAGCCCTTCTAGGAGACGACCAAGTCTACAACGTAGTCGTCACA

*Sporophila melanogaster* (**syntype**), Spomel2

Cyt-b

CTTCGGATCGCTCCTGGGCGTCTGCTTAATTACTCAAATCGTAACAGGCCTACTGCTGGCCATACACTATACAGCAGACACCAGCCTGGCCTTCTCTTCTGTCGCCCACATATGCCGAGACGTACAATTC

COI

CATCGGAACCCTATACTTAATCTTCGGCGCATGAGCCGGAATAGTAGGTACCGCCCTAAGCCTCCTCATCCGAGCAGAACTAGGCCAACCTGGAGCCCTTCTAGGAGACGACCAAGTCTACAACGTAGTCGTCACA

*Sporophila castaneiventris*, Spocas1

Cyt-b

CTTCGGGTCGCTTCTGGGCCTTTGCTTAATAACTCAAATCGTAACAGGCCTACTGCTGGCCATACACTATACAGCAGACACCAGCCTGGCCTTCTCTTCCGTCGCCCACATATGCCGAGACGTACAATTC

*Sporophila hypoxantha*, Spohyp1

Cyt-b

CTTCGGATCGCTCCTGGGCGTCTGCTTAATTACTCAAATCGTAACAGGCCTACTGCTGGCCATACACTATACAGCAGACACCAGCCTGGCCTTCTCTTCTGTCGCCCACATATGCCGAGACGTACAATTC

COI

CATCGGAACCCTATACTTAATCTTCGGCGCATGAGCCGGAATAGTAGGTACCGCCCTAAGCCTCCTCATCCGAGCAGAACTAGGCCAACCTGGAGCCCTTCTAGAAGACGACCAAGTCTACAACGTAGTCGTCACA

*Sporophila lineola*, Spolin1

Cyt-b

CTTCGGGTCATTACTGGGCCTTTGCCTAATCACTCAAATCGTCACAGGCCTACTACTTGCCATACACTACACAGCAGACACTAACCTGGCCTTCTCCTCCGTTGCCCACATATGCCGAGACGTACAATTC

COI

CATTGGAACTCTGTACTTAATCTTCGGCGCATGAGCCGGAATAGTAGGTACCGCCCTAAGCCTTCTCATCCGAGCAGAACTAGGTCAACCTGGAGCTCTCCTTGGAGACGACCAAGTCTACAACGTAGTCGTCACA

*Sporophila albogularis*, Spoalb1

Cyt-b

CTTCGGATCATTACTGGGTGTCTGCTTAATTACTCAAATCGTAACAGGCCTGCTGCTGGCCATACACTATACAGCAGACACCAGCCTGGCCTTCTCCTCCGTCGCCCACATATGCCGAGACGTACAATTC

COI

CATTGGAACCCTGTACCTAATCTTTGGCGCATGAGCCGGGATGGTAGGTACCGCCCTAAGCCTCCTTATCCGGGCAGAACTAGGCCAACCTGGAGCCCTCCTCGGAGACGATCAAGTCTACAACGTAGTCGTCACA

*Sporophila m. minuta*, Spominmin1

COI

CATTGGAACCCTATACTTAATCTTCGGCGCATGAGCCGGAATAGTAGGTACCGCCCTAAGCCTCCTCATCCGAGCAGAACTAGGCCAACCTGGAGCTCTTCTAGGAGACGACCAAGTCTACAACGTAGTCGTTACA

*Sporophila m. minuta*, Spominmin2

COI

CATTGGAACCCTATACTTAATCTTCGGCGCATGAGCCGGAATAGTAGGTACCGCCCTAAGCCTCCTCATCCGAGCAGAACTAGGCCAACCTGGAGCTCTTCTAGGAGACGACCAAGTCTACAACGTAGTCGTTACA

*Sporophila luctuosa*, Spoluc1

COI

CATTGGAACCCTGTACCTAATTTTCGGCGCATGAGCCGGAATAGTAGGTACCGCCCTAAGCCTCCTTATCCGGGCAGAGCTAGGCCAACCTGGAGCCCTCCTCGGAGACGACCAAGTTTACAACGTAGTCGTTACA
